# Genome of the fatal tapeworm *Sparganum proliferum* uncovers mechanisms for cryptic life cycle and aberrant larval proliferation

**DOI:** 10.1038/s42003-021-02160-8

**Published:** 2021-05-31

**Authors:** Taisei Kikuchi, Mehmet Dayi, Vicky L. Hunt, Kenji Ishiwata, Atsushi Toyoda, Asuka Kounosu, Simo Sun, Yasunobu Maeda, Yoko Kondo, Belkisyole Alarcon de Noya, Oscar Noya, Somei Kojima, Toshiaki Kuramochi, Haruhiko Maruyama

**Affiliations:** 1 Faculty of Medicine, University of Miyazaki, Miyazaki, Japan; 2 Forestry Vocational School, Duzce University, Duzce, Turkey; 3 Department of Biology and Biochemistry, University of Bath, Bath, UK; 4 Department of Tropical Medicine, The Jikei University School of Medicine, Tokyo, Japan; 5 Comparative Genomics Laboratory, Department of Genomics and Evolutionary Biology, National Institute of Genetics, Mishima, Shizuoka, Japan; 6 Division of Medical Zoology, Department of Microbiology and Immunology, Faculty of Medicine, Tottori University, Yonago, Japan; 7 Institute of Tropical Medicine, Central University of Venezuela, Maracay, Caracas, Venezuela; 8 Department of Clinical Laboratory Medicine, Chiba-Nishi General Hospital, Matsudo City, Chiba, Japan; 9 Department of Zoology, National Museum of Nature and Science, Tsukuba, Ibaraki, Japan

**Keywords:** Parasite genomics, Genome

## Abstract

The cryptic parasite *Sparganum proliferum* proliferates in humans and invades tissues and organs. Only scattered cases have been reported, but *S. proliferum* infection is always fatal. However, *S. proliferum*’s phylogeny and life cycle remain enigmatic. To investigate the phylogenetic relationships between *S. proliferum* and other cestode species, and to examine the mechanisms underlying pathogenicity, we sequenced the entire genomes of *S. proliferum* and a closely related non–life-threatening tapeworm *Spirometra erinaceieuropaei*. Additionally, we performed larvae transcriptome analyses of *S. proliferum* plerocercoid to identify genes involved in asexual reproduction in the host. The genome sequences confirmed that the *S. proliferum* has experienced a clearly distinct evolutionary history from *S. erinaceieuropaei*. Moreover, we found that nonordinal extracellular matrix coordination allows asexual reproduction in the host, and loss of sexual maturity in *S. proliferum* are responsible for its fatal pathogenicity to humans. Our high-quality reference genome sequences should be valuable for future studies of pseudophyllidean tapeworm biology and parasitism.

## Abstract (web summary)

Kikuchi et al. sequence the genome of the fatal tapeworm *Sparganum proliferum* and a closely related non–life-threatening tapeworm *Spirometra erinaceieuropaei*, and describe its genomic features suggesting the natural history and molecular mechanisms underlying pathogenicity. Their findings indicate that nonordinal extracellular matrix coordination is important for its asexual reproduction, and suggest that loss of sexual maturity contributes to the fatal pathogenicity of *S. proliferum* to humans.

## Introduction

The cryptic parasite *Sparganum proliferum* was first identified in a 33-year-old woman in Tokyo in 1904. The patient’s skin was infected with numerous residing cestode larva of unknown taxonomy. Ijima et al.^1^ originally designated the parasite as *Plerocercoides prolifer*, and considered it a pseudophyllidean tapeworm in the plerocercoid larval stage. In 1907, an extremely similar human case was reported by Stiles in Florida, USA, and the responsible parasite was renamed *S. proliferum*^2^. Clinical symptoms and post-mortem findings indicate that *S. proliferum* proliferates in humans and invades various organs and tissues, including the skin, body walls, lungs, abdominal viscera, lymph nodes, blood vessels, and the central nervous system, leading to miserable disease prognosis^3,4^. Not many cases have been reported to date but the infection was fatal in all reported cases (reviewed in ref. ^5^).

There was a postulation about the origin of this parasite. Some parasitologists considered it to be a new species of pseudophyllidean tapeworm, whereas others suspected that *S. proliferum* was a virus-infected or aberrant form of *Spirometra erinaceieuropaei*, based on morphological similarities^6,7^. DNA sequence analyses of mitochondrial NADH dehydrogenase subunit III, mitochondrial transfer RNA (tRNA), cytochrome oxidase subunit I, and nuclear succinate dehydrogenase iron-sulfur protein subunit (*sdhB*) genes suggested that *S. proliferum* is a closely related but distinct species to *S. erinaceieuropaei*^8,9^. However, the adult stage of *S. proliferum* has not been observed and the precise taxonomic relationships of *S. proliferum* with other worms remain unclear because most of the submitted sequences are of mitochondria origin and include only a limited number of genes. Recently, adult forms of tapeworms were isolated from wild felids and reported that they showed high-DNA sequence similarity to *S. sparganum*^10^. However, this study was based on only a few mitochondrial genes and more evidence is required to determine the precise taxonomic classification of this parasite.

In addition to taxonomic considerations, the pathogenicity of *S. proliferum* and its mechanisms of proliferation and invasion in mammalian hosts are of considerable interest. In principle, plerocercoids of pseudophyllidean tapeworms (spargana), including those of *S. erinaceieuropaei* and other *Spirometra* species, do not proliferate asexually, but migrate through subcutaneous connective tissues, causing only non-life threatening sparganosis (non-proliferative sparganosis). Other organs, such as the lungs and liver or the central nervous system, may be niches for these worms but are not commonly described. Symptoms of non-proliferative sparganosis are mainly caused by the simple mass effect^5^. The life cycles of other pseudophyllidean species including those belonging to the genera *Dibothriocephalus*, *Diplogonoporus* and *Schistocephalus* are analogous to *Spirometra* spp.^11^, except for the spectrum of second intermediate hosts, and asexual proliferation of plerocercoids, which have not previously been reported. Asexual proliferation of larvae and the destruction of host tissues are characteristic of cyclophyllidean tapeworms such as *Echinococcus*, which proliferates asexually by generating a characteristic germinative layer in a hydatid cyst form^12,13^. In another cyclophyllidean tapeworm, *Mesocestoides*, asexual multiplication is achieved by longitudinal fission^12,14^. In contrast, the pseudophyllidean *Sparganum* plerocercoid undergoes continuous branching and budding after invading the human body by an unidentified route, and produces vast numbers of progeny plerocercoids.

To clarify the phylogenetic relationship of the enigmatic parasite *S. proliferum* with other cestode species and investigate the underlying pathogenic mechanisms, we sequenced its entire genome as well as that of newly isolated *S. erinaceieuropaei* (it should be noted that recent studies suggested that Asian *S. erinaceieuropaei* can be taxonomically distinguished from European *S. erinaceieuropaei*^15,16^, but we retain the name *S. erinaceieuropaei* for the Japanese isolate). We also performed transcriptome analyses of *S. proliferum* plerocercoid larvae to identify genes that are involved in asexual proliferation in the host. Those analyses revealed its phylogeny and gene evolution that contribute to the proliferation and pathogenicity of *S. proliferum*.

## Results

### Genomic features of *S. proliferum* and *S. erinaceieuropaei*

We sequenced the *S. proliferum* genome using multiple insert-length sequence libraries (Supplementary Table 1) and compiled a 653.4-Mb assembly of 7388 scaffolds with N50 of 1.2 Mb. The *S. erinaceieuropaei* genome was assembled into 796 Mb comprising 5723 scaffolds with N50 of 821 kb. These assembly sizes were 51.9% and 63.2% of the previously published *S. erinaceieuropaei* genome (UK isolate)^17^. CEGMA and BUSCO report the percentage of highly conserved eukaryotic gene families that are present as full or partial genes in assemblies and nearly 100% of core gene families are expected in most eukaryote genomes. BUSCO analyses showed that 88.1% and 88.5% of core gene families were represent in *S. proliferum* and *S. erinaceieuropaei* genome assemblies, respectively, higher than or comparable to other previously published tapeworm genomes (Table 1). CEGMA completeness values for *S. proliferum* and *S. erinaceieuropaei* were slightly lower than those from BUSCO analyses. Low CEGMA completeness was also seen in other pseudophyllidea tapeworm genomes, including *S. erinaceieuropaei* UK isolate, *Dibothriocephalus latus*, and *Schistocephalus solidus* (Table 1 and Supplementary Data 1). Low CEGMA completeness values of these two genome assemblies could indicate pseudophyllidean-specific loss or high sequence/structure divergence of the genes that are conserved in other eukaryotic taxa. The percentage of duplicated genes in BUSCO analyses and average numbers of CEGs (CEGMA core genes) were 1.2% and 2.8% for *S. proliferum*, and 1.3% and 10.5% for *S. erinaceieuropaei*, respectively. To generate the genome assemblies we used the Haplomerger tool^18^ to collapse heterozygous haplotypes, which reduced the assembly sizes from 717.0 to 653.4 Mb and 968.1 to 796.0 Mb for *S. proliferum* and *S. erinaceieuropaei*, respectively. However, K-mer analyses of Illumina short reads estimated haploid genome sizes of 582.9 and 530.1 Mb for the two species (Supplementary Fig. 1a). These results indicate that the final assemblies roughly represent the haploid genome sizes of these tapeworms, but they still contain some heterozygous haplotypes and/or overestimated gap sizes especially in *S. erinaceieuropaei*. Ploidies were inferred from heterozygous K-mer pairs and were diploid for both species (Supplementary Fig. 1b).

**Table 1:** Statistics of genome assemblies.

	*Sparganum proliferum* (v2.2)	*Spirometra erinaceieuropaei* (v2.0)	Spirometra erinaceieuropaei (UK) (WBPS13)	*Dibothriocephalus latus* (WBPS13)	*Schistocephalus solidus* (WBPS13)	*Hymenolepis microstoma* (WBPS15)^a^	*Taenia solium* (WBPS13)	*Echinococcus multilocularis* (WBPS15)^a^
Assembly size (Mb)	653.4	796.0	1258.7	531.4	539.4	168.9	122.4	115.0
Num. scaffolds	7388	5723	482,608	140,336	56,778	7	11,237	1217
Average (kb)	88.4	139.0	2.6	3.8	10.0	24,134	11.2	94.5
Largest scaff (kb)	8099	5490	90	80	595	43,032	740	20,116
N50 (kb)	1242	821	5	7	32	25,815	68	13,762
N90 (kb)	110	167	1	2	5	17,547	5	2924
Gaps (kb)	51,020	77,788	128,163	38,407	22,091	6487	164	16,567
Cegma completeness complete/partial (%)	61.7/81.5	58.5/80.2	29.4/45.9	49.6/65.3	76.6/87.9	88.7/88.7	87.1/90.7	93.5/93.5
Average CEG number complete/partial	1.1/1.2	1.1/1.3	1.8/2.2	1.5/1.6	1.2/1.3	1.1/1.1	1.2/1.2	1.1/1.1
BUSCO completeness (Metazoa dataset/Eukaryota dataset) % of duplicated BUSCOs	72.0/88.1 2.8	71.9/88.5 10.5	33.6/37.3 14.9	38.1/53.8 1.7	70.3/86.2 2.0	79.3/n.t.^b^ 0.9	72.6/85.5 3.3	76.2/n.t.^b^1.6
Num. coding genes	18,919	22,162	39,039	19,648	18,323	10,134	12,304	10,663
Coding gene size (median; aa)	579.0	554.0	198.0	261.0	472.0	838.0	613.0	725.0
Num. transposon proteins	4124	6128	518	318	1905	58	177	83

^a^WormBase ParaSite Release 13 (WBPS13) was used for the analyses in the paper.

^b^Not tested.

The genomes of *S. proliferum* and *S. erinaceieuropaei* are highly repetitive, with ~53% of the total assembly length composed of repeats in both genomes (Supplementary Fig. 2 and Table 2). Long-interspersed nuclear elements (LINEs) occupy ~30% for both *S. proliferum* and *S. erinaceieuropaei*. These LINEs predominantly comprise the three types (Penelope, RTE-BovB, and CR1), which are also abundant in other pseudophyllidea genomes (Supplementary Fig. 2).

**Table 2:** Statistics of repeats in genomes.

	*Sparganum proliferum (v2.2)*		*Spirometra erinaceieuropaei (v2.0)*	
	Num element	% in bp	Num element	% in bp
SINEs:	36,816	1.24	35,223	1.09
LINEs:	601,034	31.63	653,492	31.49
LINE/Penelope	197,550	9.95	198,272	10.41
LINE/RTE-BovB	211,620	10.47	192,085	11.18
LINE/CR1	164,121	10.11	40,013	9.37
LTR element:1	115,379	4.93	152,710	6.49
LTR/Gypsy	89,911	3.79	52,938	1.81
DNA element:	41,513	1.75	125,893	4.07
DNA/CMC-EnSpm	4908	0.29	48,074	0.69
DNA/TcMar-Tc1	14,579	0.63	4007	0.53
Small RNA:	1964	0.09	4603	0.14
Satellites:	12,814	0.62	4931	0.17
Simple repeat:	85,865	1.60	72,020	0.41
Low complexity:	3278	0.03	3945	0.03
Unclassified:	416,273	21.10	507,009	21.55
TOTAL	1,314,936 (52.94%)		1,559,826 (52.87%)	

A total of 18,919 genes were predicted in *S. proliferum* assemblies, about 3,000 fewer than for *S. erinaceieuropaei* (22,162), which is a similar number to the number of genes in other pseudophyllidea genomes, but greater than the cyclophilidea genomes (Table 1). In studies of the *S. erinaceieuropaei* UK isolate^17^, the gene number (>39,000) was likely overestimated due to fragmentation and redundancy in the assembly. In addition, we predicted ~4,000 and ~6,000 transposon proteins (e.g., gag-pol polyproteins and reverse transcriptases) for the *S. proliferum* and *S. erinaceieuropaei* assemblies. We cannot compare these numbers directly with those of other cestode genome assemblies due to differences in gene prediction strategies, but those numbers are considerably high compared to other cestode genomes (Table 1), which is consistent with the results that *S. proliferum* and *S. erinaceieuropaei* genome are highly repetitive. Further, the presence of a large number of intact coding sequences for transposon proteins indicates that those transposable elements are still active or were active until recently in the cestode genomes.

### Phylogenetic placement of *S. proliferum*

**Fig. 1:**
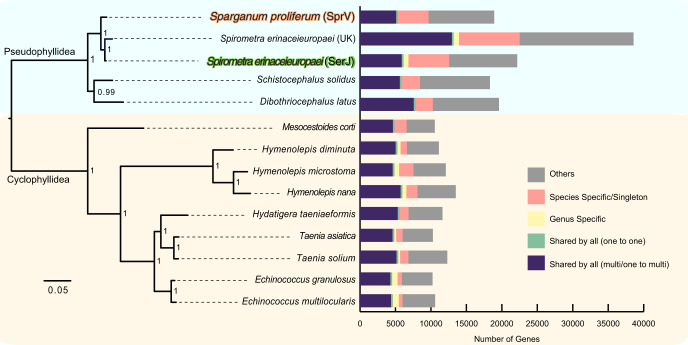
Phylogeny and gene contents of selected tapeworms. Phylogenetic relationships of *S. proliferum* and other tapeworms were inferred by a maximum-likehood tree analysis using 205 single-copy orthologous. Bootstrap confidence values via 500 replicates were shown at each node. Genes are categorised in a stack bar, and the length of stack bar is proportional to number of genes.

Phylogenetic relationships of *S. proliferum* with other cestode species were inferred from 205 single-copy orthologues (Fig. 1 and Supplementary Table 3). A clear separation was identified between pseudophyllidea and cyclophilidea clades. In the pseudophyllidea clade, *S. proliferum* occupied the basal position of the *Spirometra* cluster, in which two *S. erinaceieuropaei* isolates (Japan and UK isolates) were placed beside each other.

Phylogenetic tree topology based on mitogenomes of the 14 cestodes and all available mitogenome data of *Spirometra* in the GenBank, was similar to that of the nuclear genome (Supplementary Fig. 3 and Supplementary Table 2). Yet in contrast with the nuclear genome tree, the *S. erinaceieuropaei* UK isolate was located in a basal position of the *Spirometra* cluster, placing *S. proliferum* in the middle of *Spirometra* species, albeit with a long branch. These inconsistencies between nuclear and mitogenome trees may reflect uncertainties of species classification in the genus *Spirometra*^19,20^. Moreover, mitochondrial sequences can give poor inferences of species trees^21^. Cumulatively, these results suggest that *S. proliferum* has a close phylogenetic relationship with *Spirometra* but is clearly distinguished by genomic features and gene contents.

### Gene family evolution

**Fig. 2:**
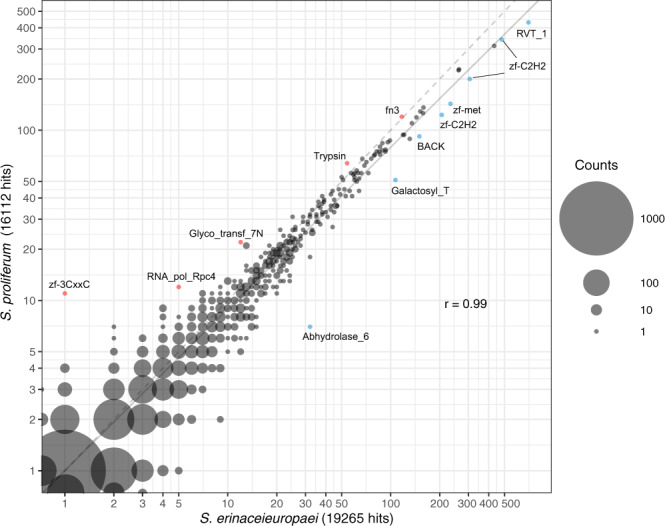
A scatterplot showing the abundance of Pfam domains in *S. proliferum* and *S. erinaceieuropaei* genomes. The size of a circle represents the number of Pfam domains. Pfam domains that are more enriched in *S. proliferum* than in *S. erinaceieuropaei* are highlighted in red. Those enriched in *S. erinaceieuropaei* relative to *S. proliferum* are highlighted in blue.

Protein family (Pfam) analyses revealed highly similar protein domain distributions of *S. proliferum* and *Spirometra* genomes (*r* = 0.99; Fig. 2 and Supplementary Data 2), confirming the close phylogenetic relationship between the two species. Only a few domains differed significantly in abundance between the two species. Among these, the *S. proliferum* genome was underrepresented in zinc-finger families (Zf-C2H2 and Zf-met), reverse transcriptase (RVT_1), galactosyltransferase, and alpha/beta hydrolase (Abhydrolase_6). Overrepresented Pfam domains in *S. proliferum* included a distinct type of zinc-finger domain (zf-3CxxC), fibronectin type III (fn3), trypsin, RNA polymerase III RPC4 (RNA_pol_Rpc4), and an N-terminal region of glycosyl transferase group 7 (Glyco_transf_7N).

**Fig. 3:**
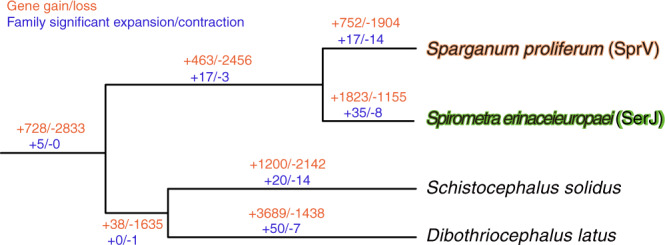
Gene family evolution of selected cestode species was inferred using computational analysis of gene family evolution (CAFE). Numbers on each branch (or lineage) indicate specific gains/losses of that branch (or lineage).

We then performed gene family analysis using OrthoFinder^22^ with the predicted proteomes of *S. proliferum*, *S. erinaceieuropaei*, and 12 other selected cestode genomes. A total of 219,816 proteins from 14 cestode species were placed into 18,738 gene families and 26,157 unassigned singletons (Fig. 1). The *S. proliferum* proteome (18,919 proteins) was encoded by 9119 gene families, among which 3674 gene families (composed of 5347 proteins) were shared by all 14 cestodes, and 4304 proteins were specific to the species or singletons. The *S. erinaceieuropaei* proteome (22,162 proteins) was clustered into 9,655 gene families. Although the *S. erinaceieuropaei* shared 7639 gene families with the other strain of *S. erinaceieuropaei* (UK), 30.4% and 39.7% of the total proteins for each strain were not shared. One of the reasons for this was the fragmented gene models in the UK genomes. To further investigate gene family evolution, we selected eight relatively high-quality genomes and performed computational analysis of gene family evolution (CAFE) to estimate gene family expansion and contraction. The analysis identified gene families with significantly higher than expected rates of gains and losses (Fig. 3 and Supplementary Table 3). Seventeen gene families were significantly expanded in the *S. proliferum* lineage, and these included annotations for fibronectin, trypsin tetraspanin family, Ras, zinc-finger C2H2 type, and core histone (Supplementary Data 3). Significantly contracted gene families (14 families) had annotations relating to signal transduction proteins, such as phosphatases and kinases (Supplementary Data 4). Fibronectin and zinc-finger C2H2 type were present in expanded and contracted families.

In the *S. erinaceieuropaei* lineage, 35 and 8 gene families were significantly expanded or contracted (Supplementary Data 5 and 6), respectively. Among them, highly lineage-specific expansion was found for 12 families (i.e., 6 or more genes in *S. erinaceieuropaei*, whereas one or no genes in *S. proliferum*. Those gene families mostly encode proteins of unknown function, but they were likely expanded after speciation from *S. proliferum* and *S. erinaceieuropaei* and may have specific roles in the *S. erinaceieuropaei* life cycle or parasitism.

### Conserved developmental pathway genes

Homeobox transcription factors are involved in patterning of body plans in animals. The homeobox gene numbers are much fewer in parasitic flatworms than in most other bilaterian invertebrates, which have a conserved set of ~100 homeobox genes. Genome sequencing of four cyclophyllid cestodes revealed that out of 96 homeobox gene families that are thought to have existed at the origin of the bilateria, 24 are not present in cestodes^23^. We identified and manually curated homeobox genes in the *S. proliferum* and *S. erinaceieuropaei* and found that the two pseudophyllid cestodes have similar homeobox class repertoires as those in cyclophyllid cestodes, in which class ANTP was the most abundant, followed by classes PRD and TALE; Table 3). The total numbers of homeobox domains identified in *S. proliferum* and *S. erinaceieuropaei* are 64 and 71, respectively, which are the most reduced of any studied bilaterian animal as these were fewer than in the cyclophyllids *Echinococcus multilocularis* and *Taenia solium* (Table 3). The three homeobox families Pou/Pou6, ANTP/Bsx, and ANTP/Meox were not present in *S. proliferum* and *S. erinaceieuropaei*, whereas the homeobox family ANTP/Ro was found in *S. proliferum* and *S. erinaceieuropaei* but not in *E. multilocularis* and *T. solium* (Supplementary Fig. 4).

**Table 3:** Homeobox complement in *S. proliferum* and *S. erinaceieuropaei* compared with other tapeworms and bilaterians.

Homeobox class	*Sparganum proliferum* (v2.2)	*Spirometra erinaceieuropaei* (v2.0)	*Taenia solium* (WBPS13)	*Echinococcus multilocularis* (WBPS13)	*Branchiostoma floridae*
ANTP	25	30	36	25	58
PRD	10	8	11	15(18)	21
CUT	3	4	3	3	4
SINE	3	4	2	3	3
TALE	8	10	11	12	10
CERS	2	1	2	2	1
POU	3	4	4	5	6
LIM	6	6	7	7	8
ZF	4	4	3(2)	3(2)	4
Total	64	71	79	75	115

Comparisons between *S. proliferum* and *S. erinaceieuropaei* showed that the homeobox families TALE/Pknox, ANTP/Hox1, ANTP/Msxlx, and POU/Pou-like are missing in *S. proliferum*, despite being present in the other cestodes. In contrast, the homeobox families ANTP/Dbx and PRD/Alx were found in *S. proliferum* but not in *S. erinaceieuropaei*.

Other conserved genes with roles in flatworm developmental pathways, such as Hedgehog and Notch, were largely conserved in *S. proliferum* and *S. erinaceieuropaei*. However, in the Wnt pathway, whose complement is much smaller than the ancestral complement in tapeworms^23^, two further genes (Axin and LEF1/TCF) were missing in *S. proliferum* and *S. erinaceieuropaei* (Supplementary Data 7).

### Horizontally transferred genes

To determine whether the present genomes contained horizontally transferred genes (HTGs) from other organisms, we used a genome-wide prediction method based on a lineage probability index using the software Darkhorse2^24^ and identified 19 and 33 putative HTGs in *S. proliferum* and *S. erinaceieuropaei*, respectively (Supplementary Data 8 and 9). For these transfers, all possible host organisms were bacteria except for one *Spirometra* gene that has high similarity to a chlorella virus gene. Orthologues of most *S. proliferum* putative HTGs were also detected as horizontally transferred in *S. erinaceieuropaei*. Moreover, possible host bacteria, including *Marinifilum breve*, *Aphanizomenon flos-aquae*, *Alcanivorax* sp., and *Vibrio* sp., were shared by the two cestode species and were aquatic or marine bacteria, indicating that these genes were acquired by a common ancestor of the two tapeworms, which had aquatic phase in the life cycle.

### Signatures of selection in the *S. proliferum* lineage

Positive selection is a mechanism by which new advantageous genetic variants sweep through a population and drive adaptive evolution. To investigate the roles of positive selection in the evolution of *S. proliferum* from a non–life-threatening ancestor, we performed d*N*/d*S* branch-site model analyses with 755 single-copy orthologous genes from 12 tapeworms (Supplementary Data 10) and identified a total of 35 positively selected genes in the *S. proliferum* lineage (Supplementary Table 4). Evolutionary pressures were identified for some genes that are essential to cellular processes, including transcription/RNA processing/translation genes encoding DNA-directed RNA polymerase II subunit, polypyrimidine tract-binding protein, adenylate kinase, ribosomal protein L21, snu13 NHP2-like protein, and eukaryotic translation initiation factors. Other identified genes were related to transportation (dynein intermediate chain 2) and mitochondrial processes (Rieske). Genes involved in stress and immune responses, such as DNAJ/Hsp40, HIKESHI protein, Toll-like receptor, and Ig_3/Ig, were also positively selected in the *S. proliferum* lineage, along with the RAS oncogene *Rab-4A*.

Circumstances where environmental change eliminates or weakens selection pressures formerly important for the maintenance of a particular trait, have been termed “relaxed selection”^25^. Such circumstances include, for example, eyesight in lightless caves and pathogen resistance after disappearance of pathogens. Genes under relaxed selection can be detected using the ratio of non-synonymous to synonymous substitutions and a comparison in a phylogenetic framework^26^. In *S. proliferum* we sought to test the relaxed selection that may have acted on genes involved in development or sex reproduction under its contentious asexual reproduction. Using the alignment set used in the positive selection tests, we detected 10 genes that were under “relaxed selection” in the *S. proliferum* lineage, relative to the other tapeworm lineages (Supplementary Table 5). These genes encode proteins with putative roles in developmental regulation and cell differentiation. In particular, the receptor roundabout (ROBO) and secreted molecules of the SLIT family, together, play important roles in guiding axons and proper morphogenesis^27^. The Rho-GTPase-activating protein is also highly expressed in highly differentiated tissues and affects cell differentiation by negatively regulating Rho-GTPase signalling^28^. Delta-like protein (DLL) is an inhibitory ligand of the Notch receptor pathway and is expressed during brain development^29^. Vascular endothelial growth factor receptor is also known to regulate stem cell homeostasis and repopulation in planarian species^30^. Hence, these instances of relaxed selection indicate that certain developmental pathways became less important for the worm or, further, possibly the worm has long since used those pathways. We also identified two genes encoding cadherin (protocadherin) that were subject to relaxed selection. Cadherein is a transmembrane protein that mediates cell–cell adhesion in animals and the signatures of relaxed selection suggest cell adhesion processes have diverged between the worm species.

### Differential gene expression involved in asexual proliferation and parasitism

**Fig. 4:**
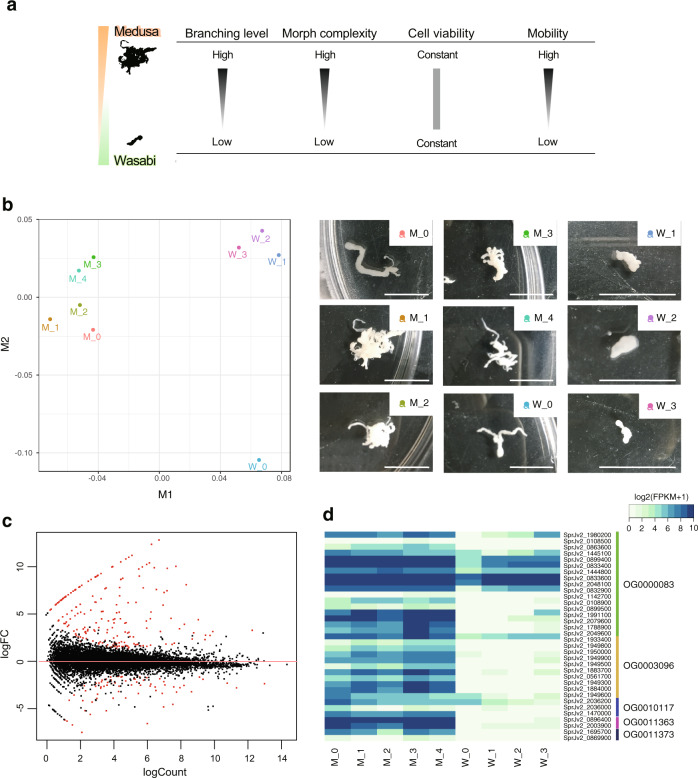
Comparison of gene expression in highly branching worms (medusa form) relative to static worms (wasabi form) of *S. proliferum*. **a** A schematic illustration to show characteristics of medusa and wasabi forms of plerocercoid. **b** Multidimensional scaling (MDS) analyses of RNA-seq samples clearly separate the two forms by dimension 1. Pictures of used samples are shown on the right. Scale bars indicate 10 mm. **c** Bland-Altman (MA) plot of the two-form comparison; dots represent transcripts and log2-fold-changes (medusa/wasabi) plotted against average abundance in counts per million. Red dots indicate differentially expressed transcripts with false discovery rates (FDR) of <0.05 and fold-changes of >2. **d** Heatmap of gene families encoding novel secreted proteins; the heat map shows log2 fragments per kilobase per million reads mapped (FPKM) values for five gene families.

We maintained *S. proliferum* via serial infection of mice and found that some plerocercoid worms exhibit a highly branching structure, which was observed frequently in heavily infected mice. In contrast, in mice with low worm burdens, most worms had unadorned non-branching morphology. To investigate physiological characteristics of those worms, we compared morphology, viability and mobility of 81 worms showing variable branching levels with branch numbers ranging from 1 to 79 (Supplementary Table 6). We calculated fractal dimension value for each worm, which represents morphological complexity, and found that the value better represents the worm status than using only the branch numbers. Fractal dimension not only shows a good correlation with the number of branches (*r* = 0.59, *p*-value = 6e-9) (Supplementary Fig. 5a), but is also possible to distinguish the unadorned forms representing low branching activity from worms recently split from a highly branching worm, which shows simple but relatively slender forms. Cell viability evaluated with the assays based on cellular NADH/NADPH activity^31^ and extracellular lactate dehydrogenase (LDH) activity^32^ showed almost no correlation with the fractal dimension values (*r* = −0.01, *p*-value=0.88 and *r* = 0.08, *p*-value=0.51, respectively) (Supplementary Figure 5b and S5c). However, higher mobility was observed in the high-complexity worms than the low ones (*r* = 0.28, *p*-value = 0.04), which possibly results in more frequent fragmentation of branches (Supplementary Fig. 5d). Thus, those high-complexity worms are considered the main sources of new plerocercoid worms in the host via their frequent branching and high mobility. We call this highly branching form “medusa-form” and the unadorned non-branching form “wasabi-form” because of the resemblances of those structures with Medusa’s head” and wasabi rhizomes (Fig. 4a). Further histological observations revealed that the parenchymal cavity, which is characteristic of *S. proliferum*^33^, was more developed in the medusa-form and the cavities were often filled with contents stained in pink by hematoxylin and eosin (H-E) (Supplementary Fig. 6). In contrast, most parenchymal cavities in the wasabi-form were empty. Additionally, in the medusa-form, we found loci where nuclei were concentrated. Calcareous bodies, demonstrated by von Kossa staining, were more often observed in the medusa-form than the wasabi-form. These observations suggest that the typical histological characteristics of *S. proliferum* reported previously were of the medusa-form^33^.

We performed a transcriptome analysis using nine RNAseq libraries generated from five and four worms showing typical morphological characters of medusa and wasabi forms, respectively, to identify genes involved in the plerocercoid proliferation. Interestingly, RNA expression patterns of the two forms were clearly separated by the first dimension of Multidimensional scaling (MDS) plot based on Spearman correlations (Fig. 4b and Supplementary Table 7). EdgeR RNAseq comparisons identified 357 differentially expressed genes (DE genes) between the medusa and wasabi forms (246 upregulated and 111 downregulated in medusa forms) (Fig. 4c). In this analyses we used a total of 23,043 gene models which included 4124 transposon proteins and found many transposon proteins such as gag-pol polyproteins and reverse transcriptases (36 genes) were upregulated in the medusa form. Other than that, the upregulated set in medusa forms were dominated by genes encoding peptidases and peptidase inhibitors, such as tolloid-like proteins (19 genes), chymotrypsin-like proteins (6 genes) and CAP domain-containing proteins (12 genes) (Supplementary Data 11). This set of DE genes was enriched in the GO categories for metalloendopeptidase activity and proteolysis (Supplementary Table 8). Downregulated genes also encoded a variety of peptidases and peptidase inhibitors, including leucyl aminopeptidase (five genes), chymotrypsin-like proteins (seven genes), and kunitz bovine pancreatic trypsin inhibitor domain protein (three genes), with high representation under the GO terms metalloexopeptidase, aminopeptidase, and manganese ion binding (Supplementary Table 8). Peptidases and peptidase inhibitors are secreted by many types of pathogens, including bacteria, fungi, and parasites, and often play critical roles in survival and virulence^34–36^. Other genes known to be involved in pathogenicity in other pathogens were also upregulated in the medusa form, including genes encoding multidrug resistance-associated proteins^37^ and tetraspanins. The latter proteins have four transmembrane domains and not only play roles in a various aspects of cell biology but also are used by several pathogens for infection and regulate cancer progression^38^.

Genes that are involved in cell-growth and cancer development were also upregulated in the medusa form, including those encoding proteins from wnt (WNT-111 and WNT-5) and ras/rab (RAS-0b, RAS-2 and Rasef) pathways, transcription factors/receptors (SOX1a, fibroblast growth factor receptor) and homeobox proteins (prospero, PAX, orthopedia ALX and ISL2).

It has been shown that expansions of gene families and changes in expression levels have been associated with the evolution of parasitism in previous studies^39,40^. An upregulation of genes from expanded gene families was also found in *S. proliferum*. For instance, 12 out of 20 genes were identified as upregulated from an expanded gene family encoding CAP domain-containing proteins (orthogroup OG0000040). The orthogroup OG0000044 includes 49 *S. proliferum* genes encoding Chymotrypsin-like (mastin-like) proteins (Supplementary Data 3), and six of these were upregulated and another six were downregulated in the medusa form (Supplementary Data 11). Gene family OG0000044 is Pseudophillidea specific and gene family expansions were observed in *Dibothriocephalus*, *Spirometra* and *S. proliferum* although a phylogenetic analysis of these genes could not define upregulated or downregulated gene cluster in the phylogenetic tree (Supplementary Fig. 7). Phylogenetic analyses of the other expanded gene families containing multiple DE genes indicate that some of these orthogroups are conserved across flatworms, while others are specific to the Pseudophillidea clade of flatworms (Supplementary Fig. 7).

Thirty-six upregulated genes showed fold-changes differences >200 (Supplementary Data 11), including eight genes encoding tolloid-like proteins. In mammals, tolloid-like proteins compose a small group called BMP-1/TLD family with bone morphogenetic proteins (BMP), playing fundamental roles in morphogenesis and extracellular matrix (ECM) assembly via a direct effect on growth factors such as TGF-β and IGFs^41,42^. The high fold-change of the tolloid-like protein genes in the medusa-form is, therefore, likely reflecting the high-branching activity of the worms and suggests an important role of these proteins in *S. proliferum* asexual proliferation. *SprJv2_0697700.1* gene encoding acetylcholinesterase also showed a very high fold-change (>1000 folds). Acetylcholinesterases have a well-defined role in cholinergic neurotransmission in both vertebrates and invertebrates^43^, but they are also known to be secreted by a variety of parasitic nematodes and have been speculated to work in modulating host’s immunity, inflammation and physiology^44,45^. The high expression of the gene in the medusa-form made us conceive a possibility that it plays a role in the parasitism in some manner, though no secretion signal peptides were identified in the protein and little is known about secreted acetylcholinesterases of cestodes^46^.

Notably, 24 of the 36 high fold-change genes were uncharacterised proteins. In total DE genes, 85 out of 246 that were upregulated in medusa forms have no known functions. These included 17, 10, 3, 2, and 2 genes from orthogroups OG0000083, OG0003096, OG0010117, OG0011363, and OG0011373, respectively. These orthogroups were expanded in the *S. proliferum* lineage and most DE genes had extremely high fold-changes (Fig. 4c). As their products predominantly harbour secretion signal peptides (Supplementary Data 11), they are likely to be secreted by the parasite into the host and play important roles in parasitism, aberrant larval proliferation in the host, and/or modulation of host immunity.

## Discussion

*S. proliferum* is a cryptic parasite with fatal consequences, but its phylogeny and life cycle are poorly understood. In this study, we sequenced the *S. proliferum* genome and performed comparative genomics with other tapeworm species, including the newly sequenced *S. erinaceieuropaei* genome. The *S. erinaceieuropaei* genome was sequenced previously^17^, with an estimated genome size of more than 1.2 Gb, but because the source material was from a biopsy the assembled sequence was highly fragmented. Hence, the *S. erinaceieuropaei* genome presented herein provides a more reliable estimate of the size and contents of this parasite genome although the kmer-based haploid genome size estimate suggests that the new assembly still contains un-collapsed heterozygous haplotypes. The new genome assembly was about two thirds of the size of the previous assembly, but remains the largest genome among sequenced tapeworms. Compared to cyclophyllidean tapeworms, including *Echinococcus* and *Taenia* spp., for which high-quality genome references are available^23,47,48^, genome information for pseudophyllidean tapeworms is limited^49^. The genomes presented in this study could, therefore, serve as a powerful resource for more comprehensive studies of tapeworm genomics and will facilitate the understanding of pseudophyllidean tapeworm biology and parasitism.

There have been three big knowledge gaps for the cryptic tapeworm *S. proliferum*: (1) its phylogenetic relationship with *Spirometra* species, (2) its life cycle including the definitive and intermediate hosts, and (3) genetic and physiological differences with non-proliferating *Spirometra* species that enable the worm to reproduce asexually in non-definitive hosts, such as humans and mice.

To determine phylogenetic relationships, we confirmed that the genetic sequence of *S. proliferum* is distinct from that of *S. erinaceieuropaei*, despite the close relationship between these species. Specifically, the *S. proliferum* genome is about 150-Mb smaller and contains 3000 fewer protein-coding genes than in *S. erinaceieuropaei*. Both genomes, nonetheless, showed diploidy. These data suggest that *S. proliferum* is not an aberrant form of *Spirometra* worm by virus infection or by small mutations^6,7^ and not a hybrid origin of multiple *Spirometra* species. In agreement, no virus-like sequences were detected in *S. proliferum* DNA or RNA raw reads.

We were unable to identify definitive or intermediate hosts of *S. proliferum* in the current study. Recent horizontal transfers of genes or mobile elements can indicate ecological relationships, because HGT events occur between closely associated organisms. Well-known examples include HGT from Wolbachia symbionts to their host insect^50,51^ and transfer of BovB retrotransposons between ruminants and snakes via parasitic ticks^52,53^. We found that RTE/BovB repeats are abundant in the *S. proliferum* genome, but were likely acquired by an ancestral pseudophyllidea, as indicated by their abundance in *D. latus* and *S. solidus*. Moreover, our HGT screening analyses indicate several genes were acquired from bacteria but these HGTs likely have occurred before specification of *S. proliferum* and *S. erinaceieuropaei*. The high-quality reference genomes presented herein, however, provide valuable resources for further attempts to identify vestigial *S. proliferum* sequences in other organisms or to perform analyses of protein–protein interactions between hosts and parasites. Recently, a possible adult form of *S. proliferum* was isolated from wild felids and fragments of mitochondrial cox-1, nad-1 and atp-6 genes were sequenced^10^. Although those sequences showed higher similarities to *S. proliferum* than *S. erinaceieuropaei* or other tapeworms, the percentages of identities with the *S. proliferum* mitochondrion were relatively low (93% (320/343) in cox1, 96% (284/296) in nad1 and 92% (531/577) in atp6), suggesting they are closely related but not identical species. Further sequencing analyses of those worms will help understand the origin, biology and pathogenicity of *S. proliferum*.

Loss of genes that are involved in the development of multicellular organisms and nervous systems, including homeobox genes and genes for zinc-finger domain-containing proteins, as well as relaxed selection of some developmental genes (ROBO, Slit, RHOGAP, etc.), suggests that *S. proliferum* has lost the ability to undergo proper development and complete the sexual life cycle. Although the precise functions of homeobox genes in tapeworms remain elusive, homeobox families that are missing in *S. proliferum* (TALE/Pknox, ANTP/Hox1, ANTP/Msxlx and POU/Pou-like) appear to have important roles in the development of embryos and adult body plans. For example, Hox1 of the HOX gene family specifies regions of the body plan of embryos and the head–tail axis of animals^54^. Products of the Pknox gene family, also known as the PREP gene family, are implicated as cofactors of Hox proteins^55^. M*sxlx* homeobox gene was highly upregulated in the ovaries, continually expressed in fertilised ova in the uterus in *Hymenolepis microstoma* and suggested to be related to the female reproductive system in this tapeworm^56^. POU class genes are present in all animals playing roles in neurogenesis, pluripotency, and cell-type specification. The POU homologue Oct-4 is one of the four “Yamanaka factors” used to induce pluripotent stem cells in mammals^57^. Specific loss of Pou-like genes and relaxed selection of Pou3 suggest that *S. proliferum* has low dependency on POU genes.

We contend that the loss of sexual maturity of this parasite is related to its fatal pathogenicity in humans, because survival of the parasite is dependent on asexual reproductive traits of budding and branching, which lead to 100% lethality in infected humans. Accordingly, we identified genes that are upregulated in vigorously budding worms using transcriptome analyses and then selected genes that are putatively important for asexual proliferation, such as a variety of peptidase genes and oncogene-like genes. Among them, groups of secreted proteins with unknown functions were of great interest. They were expanded in the *S. proliferum* genome and showed more than 10-fold-changes in expression levels. Recently, an *S. erinaceieuropaei* gene belonging to one of those groups (orthogroup OG0000083) was cloned and named plerocercoid-immunosuppressive factor (P-ISF)^58^. P-ISF is a cysteine-rich glycoprotein abundant in plerocercoid excretory/secretory products and likely involved in immunomodulation of its hosts, by suppressing osteoclastgenesis including the gene expression of TNF-α and IL-1β, and nitric oxide production in macrophages^59,60^. Upregulation of P-ISF genes in *S. proliferum* proliferating worms is, therefore, reasonable and the expansion of the gene family in *S. proliferum* indicates the considerable contribution to the specific life cycle. The other upregulated gene families of unknown function are also expanded in *S. proliferum*, suggesting possible important roles in the hosts, therefore, future studies of these novel genes are required to fully understand the mechanism underlying the *S. proliferum* parasitism.

Fibronectin is an extracellular matrix (ECM) glycoprotein that controls the deposition of other ECM proteins, including collagens and latent TGF-beta binding protein^61^. During branching morphogenesis, accumulations of fibronectin fibrils promote cleft formation by suppressing cadherin localisation, leading to loss of cell–cell adhesion^62^. The present observations of the *S. proliferum* lineage show specific expansions of three gene families containing fibronectin type III domains. *S. proliferum* also had fewer cadherin genes than *S. erinaceieuropaei* and three of them are subject to relaxed selection in *S. proliferum*. Furthermore, we found extremely high upregulation of tolloid-like proteins in highly branching medusa worms, which are known to be involved in ECM assembly in mammals via a direct effect on growth factors. These results collectively suggest nonordinal ECM coordination in *S. proliferum*, allowing the formation of highly branching structures and enabling asexual proliferation in the host.

## Methods

### Biological materials

*S. proliferum* strain Venezuela was used for the genome analyses. The parasite was originally isolated from a Venezuelan patient in 1981 and has been maintained by serial passages using BALB/c mice via intraperitoneal injections of the plerocercoids in National Science Museum as described in Noya and colleagues^63,64^. *S. erinaceieuropaei* used for the genome project was a natural isolate obtained from a Japanese four-striped rat snake (*Elaphe quadrivirgata*) purchased in Yamaguchi prefecture, Japan in 2014.

### DNA and RNA extraction and sequencing

*S. proliferum* worms were collected from the abdominal cavity of infected mice and washed thoroughly with 1x PBS. Plerocercoids of *S. erinaceieuropaei* were isolated from the subcutaneous tissues of the snake. Genomic DNA was extracted from a single worm using Genomic-tip (Qiagen) following the manufacturer’s instructions and used for genome sequencing for both species.

Paired-end sequencing libraries (Supplementary Table 1) were prepared using the TruSeq DNA Sample Prep kit (Illumina) according to the manufacturer’s instructions. Multiple mate-paired libraries (3, 8, 12 and 16 kb) were also constructed using the Nextera Mate-Paired Library Construction kit (Illumina). Libraries were sequenced on the Illumina HiSeq 2500 sequencer using the Illumina TruSeq PE Cluster kit v3 and TruSeq SBS kit v3 (101, 150 or 250 cycles x 2) or the Illumina MiSeq sequencer with the v3 kit (301 cycles x 2) (Supplementary Table 1). The raw sequence data were analysed using the RTA 1.12.4.2 analysis pipeline and were used for genome assembly after removal of adapter, low quality, and duplicate reads.

RNA was extracted from individual worms using TRI reagent according to the manufacturer’s instructions. Total RNA samples were qualified using Bioanalyzer 2100 (Agilent Technology, Inc.). Only samples with an RNA integrity value (RIN) greater than 8.0 were used for library construction. One hundred ng of total RNA was used to construct an Illumina sequencing library using the TruSeq RNA-seq Sample Prep kit according to the manufacturer’s recommended protocols (Illumina, San Diego, USA). The libraries were sequenced for 101 or 151 bp paired-ends on an Illumina HiSeq2500 sequencer using the standard protocol (Illumina).

### K-mer analysis

A K-mer count analysis was performed using K-mer Counter (KMC)^65^, on the paired-end Illumina data. Only the first read was used to avoid counting overlapping k-mers. Genome size and ploidy estimations were performed using Genomescope^66^ and Smudgeplot, respectively^67^.

### Genome assembly

Illumina reads from multiple paired-end and mate-pair libraries (Supplementary Table 1) were assembled using the Platanus assembler^68^ with the default parameter. Haplomerger2^18^ was then used to remove remaining haplotypic sequences in the assembly and contigs were further scaffolded using Illumina mate-pair reads using SSPACE^69^. CEGMA v2^70^ and BUSCO^71^ were used to assess the completeness of the assemblies.

Mitochondrial genomes (mitogenomes) were reconstructed from Illumina reads with MITObim version 1.6^72^. Mitochondrial fragments in the nuclear genome assembly were identified by BLASTX using *S. mansonai* mitochondrial genes as queries and those fragments were extended by iterative mappings of Illumina short reads using MITObim. Assembled mitogenomes were annotated for protein-coding, tRNA and ribosomal RNA genes using the MITOS web server^73^. Assemblies and annotations were manually curated using the Artemis genome annotation tool^74^ with based on evidence supports from sequence similarity to other published mitogenomes.

### Repeat analysis

Repeats within the genome assemblies were identified using the combined outputs of RepeatModeler (v1.0.8, http://www.repeatmasker.org/RepeatModeler.html), MITE-hunter (v11-2011)^75^, LTR-harvest/LTR-digest (v.1.6.1)^76^ and TransposonPSI (v08222010) (http://transposonpsi.sourceforge.net). For each species, usearch v7^77^ and RepeatClassifier (v1.0.8, http://www.repeatmasker.org/RepeatModeler.html) was used to cluster and classify repeat sequences from multiple tools to generate consensus sequences for a non-redundant repeat library with ≥80% identity threshold. RepeatMasker (v.3.2.8, http://www.repeatmasker.org) was then run with the custom repeat library, to calculate the distribution of each repeat and its abundance in the genome assemblies.

### Gene prediction and functional annotation

To predict protein-coding genes, Augustus (v. 3.0.1)^78^ was trained for *S. proliferum* and *S. erinaceieuropaei*, individually, based on a training set of 500 non-overlapping, manually curated genes. To obtain high-confidence curated genes, a selection of gene models from gene predictions based on Augustus *S. mansonai* parameters, were manually curated in Artemis using aligned RNA-seq data and BLAST matches against the NCBI database. RNA-seq reads were mapped to the genomes using Hisat2 (parameters: --rna-strandness RF --min-intronlen 20 –max-intronlen 10000)^79^. Based on the Hisat2 alignments, the bam2hints programme (part of the Augustus package) was used to create the intron hints, with minimum length set to 20 bp. Augustus were run with trained parameters using all the hints for that species as input. Introns starting with “AT” and ending with “AC” were allowed (--allow_hinted_splicesites=atac). A weight of 10^5^ was given to intron and exonpart hints from RNA-seq. If Augustus predicted multiple, alternatively spliced transcripts for a gene, we only kept the transcript corresponding to the longest predicted protein for further analyses. The gene prediction sensitivity and specificity at exon level evaluated with ~200 each manually curated genes (not overlapping with the training sets) were 79.0% and 64.2% for *S. proliferum* and 76.2% and 64.0% for *S. erinaceieuropaei*, respectively.

Functional annotations were performed on the gene models based on multiple pieces of evidence, including BLASTP search against NCBI nr database and the latest version Pfam search (ver. 30.0) with HMMER3^80^. Gene ontology (GO) terms were assigned to genes using Blast2Go (v2)^81^ with BLAST search against NCBI nr database and the InterProScan results. TransposonPSI (release 08222010; http://transposonpsi.sourceforge.net) was used to identify potential transposon proteins in the protein sets.

### Gene family analysis

To estimate branch or lineage-specific gain and loss of orthologous gene families, OrthoFinder^22^ and CAFÉ (v3)^82^ under parameters “-p 0.01, -r 1000” were used. The genome datasets of 12 cestode species (*Dibothriocephalus latus*^49^, *Echinococcus granulosus*^23^, *Echinococcus multilocularis*^23^, *Hymenolepis diminuta*^49^, *Hymenolepis microstoma*^83^, *Hymenolepis nana*, *Hydatigera taeniaeformis*^49^, *Mesocestoides corti*^49^, *Spirometra erinaceieuropaei*^17^, *Schistocephalus solidus*^49^, *Taenia asiatica*^84^, *Taenia solium*^23^) used for the analyses were downloaded from WormBase Parasite WBPS13 (https://parasite.wormbase.org/)^85^.

### Species tree reconstruction

Amino acid sequences in each single-copy gene family were aligned using MAFFT version v7.22152^86^, poorly aligned regions were trimmed using GBlocks v0.91b53^87^, and then the trimmed alignments were concatenated. Genetic distance was calculated with the Poisson model and uniform rates among sites. A maximum-likelihood phylogenetic tree was produced based on the concatenated alignment using RAxML v8.2.754^88^ with 500 bootstrap replicates. The best-fitting substitution model for each protein alignment was identified using the RAxML option (-m PROTGAMMAAUTO). Mitochondrial genome phylogeny was also constructed by the same method using 12 protein-coding genes on mitogenomes.

### Identification of developmental pathway genes

Homeobox genes in *S. proliferum* and *S. erinaceieuropaei* were identified using OrthoFinder results and BlastP analyses using lophotrochozoan genes as queries^89,90^. Identified genes were manually curated for the gene structures using Artemis^74^ and absence of genes was confirmed by tBlastn analyses using protein queries against the genome sequences. Genes involved in other conserved developmental pathways, including Hedgehog, Notch and Wnt were also identified in the same way^23^.

### Screening for horizontally transferred genes

To screen potential horizontal gene transfers (HGTs) into the *S. proliferum* and *S. erinaceieuropaei* lineages, we used DarkHorse v2, which detects phylogenetically atypical proteins based on phylogenetic relatedness of BlastP hits against a taxonomically diverse reference database using a taxonomically weighted distance algorithm^24^. Options (-n 1 -b 0.5 -f 0.1) were used in DarkHorse HGT screening. Franking genes of the identified candidates were checked for similarity to any cestode genes to exclude possibilities of mis-assembly or contamination.

### Positive/relax selection scans (d*N*/d*S*)

To analyse selection pressures in *S. proliferum* genes, the ETE3 Python package^91^ for CODEML^92^ was employed to calculate the non-synonymous (d*N*) and synonymous (d*S*) substitutions rates, and the ratio (d*N*/d*S* or *ω*). Nucleotide sequences of 755 single-copy orthologue genes from 12 cestode species (*S. proliferum*, *S. erinaceieuropaei*, *D. latus, S. solidus, H. diminuta, H. nana, H. taeniaeformis, T. solium, T. asiatica, E. multilocularis, E. granulosus, M. corti*) were aligned based on each corresponding amino acid alignment using Pal2aln v14^93^ with the parameters (-nomismatch and –nogap). After removing poor alignments manually, d*N*/d*S* were estimated using branch-site models with *S. proliferum* as the foreground and other branches in the tree as the background. The non-null model (bsA) were compared with the null model (bsA1) for each tree using a likelihood ratio test (LRT), where log-likelihood ratios were compared to a chi-square distribution with 1 degree of freedom. False discovery rate (FDR) correction were performed over all the *p*-values and genes showing FDR <0.05 were manually curated before obtaining final d*N*/d*S* values.

Test for relaxed selection was performed using the RELAX tool^26^ with aforementioned nucleotide alignments of the single-copy orthologue genes. The relaxation parameter *k* was calculated for each branch and tested by LRT with *S. proliferum* as foreground and the others as background, and genes showing *k*-values <1 and likelihood ratio test values <0.05 were listed as candidate genes under relaxed selection.

### Worm viability/mobility assays and histological observation

*S. proliferum* plerocercoid worms isolated from infected mice were washed with PBS and transferred RPMI media (Gibco) both prewarmed at 37 °C. Worm mobility in RPMI at 37 °C was recorded for 15 min using Wormlab instrument (MBF Bioscience) and analysed for the average migration speed using ImageJ and wrMTrck plugin^94,95^. Worm viability and cell damage were evaluated using water-soluble tetrazolium salt (WST)‐8 (Cell Counting Kit-8, Dojindo Laboratories) and lactate dehydrogenase (LDH, Cytotoxicity LDH Assay Kit-WST, Dojindo Laboratories) assays, respectively. Complexities of worm morphs were quantified by calculating fractal dimensions using ImageJ and FracLac plugin^96^. For histological observation, isolated worms were immediately fixed in 4% paraformaldehyde phosphate buffer solution and embedded in paraffin. Tissue slides of 5 μm thick sections were stained with haematoxylin and eosin (H-E) and observed under light microscopy.

### RNAseq analysis

For gene expression analyses, *S. proliferum* plerocercoid worms were grouped into two types based on the morphology and proliferation activity; worms vigorously branching to form structure like Medusa’s head and worms under static form to form like wasabi rhizomes (Fig. 4a). Five medusa-form and four wasabi-form worms were collected from infected mice on ~50 weeks post inoculation. RNA was extracted from the individual worms and sequenced independently as described above. RNAseq reads were mapped to the *S. proliferum* reference genomes (v2.2) using Hisat2^79^ (parameters: --rna-strandness RF --min-intronlen 20 –max-intronlen 10000). Mapped read count of each gene was calculated using HTSeq^97^ with options (−s no, −a 10, −m union) and differential expression analyses were performed using EdgeR v3.2.4^98^. A transcript was identified as differentially expressed in a pairwise comparison if the following criteria were met: false discovery rate (FDR) ≤ 0.001 and fold-change ≥2.0. Spearman correlation was calculated between samples using R and used to generate for multidimensional scaling (MDS) plots. FPKM values were calculated using Cufflinks packages v2.2.1^99^ and used to generate and gene expression heatmaps.

### Statistics and reproducibility

Statistical analyses were performed using independent biological replicates, which is considered to be a large enough sample for the statistical estimations, as described in Methods with available packages including R, edgeR^98^, RAxML^88^, CAFÉ^82^, DarkHorse^24^ and ETE3^91^. Reproducibility can be accomplished using our own parameters mentioned in Methods.

### Ethics approval and consent to participate

All animal experiments in this study were performed under the applicable laws and guidelines for the care and use of laboratory animals, as specified in the Fundamental Guidelines for Proper Conduct of Animal Experiment and Related Activities in Academic Research Institutions under the jurisdiction of the Ministry of Education, Culture, Sports, Science and Technology, Japan, 2006.

### Reporting summary

Further information on research design is available in the Nature Research Reporting Summary linked to this article.

## Supplementary information

Supplementary Information

Description of Additional Supplementary Files

Supplementary Data

Reporting Summary

## Data Availability

All sequence data from the genome projects have been deposited at DDBJ/ENA/GenBank under BioProject accession PRJEB35374 and PRJEB35375. All relevant data are available from the corresponding authors on reasonable request.
