# Supplementary material for: Genome of the fatal tapeworm Sparganum proliferum uncovers mechanisms for cryptic life cycle and aberrant larval proliferation
Source: Commun Biol. 2021 May 31;4:649. doi: 10.1038/s42003-021-02160-8 (PMC8166898; doi:10.1038/s42003-021-02160-8)
Supplement: Supplementary file 1 — Supplementary Information [file 42003_2021_2160_MOESM1_ESM.pdf]

Supplementary Materials for

Genome of the fatal tapeworm *Sparganum proliferum* uncovers mechanisms for cryptic life cycle and aberrant larval proliferation

Taisei Kikuchi, Mehmet Dayi, Vicky L. Hunt, Kenji Ishiwata, Atsushi Toyoda, Asuka Kounosu, Simo Sun, Yasunobu Maeda, Yoko Kondo, Belkisyole Alarcon de Noya, Oscar Noya, Somei Kojima, Toshiaki Kuramochi, Haruhiko Maruyama

correspondence to: taisei_kikuchi@med.miyazaki-u.ac.jp

This PDF file includes:

Supplementary Figure 1 to 7

Supplementary Table 1 to 8

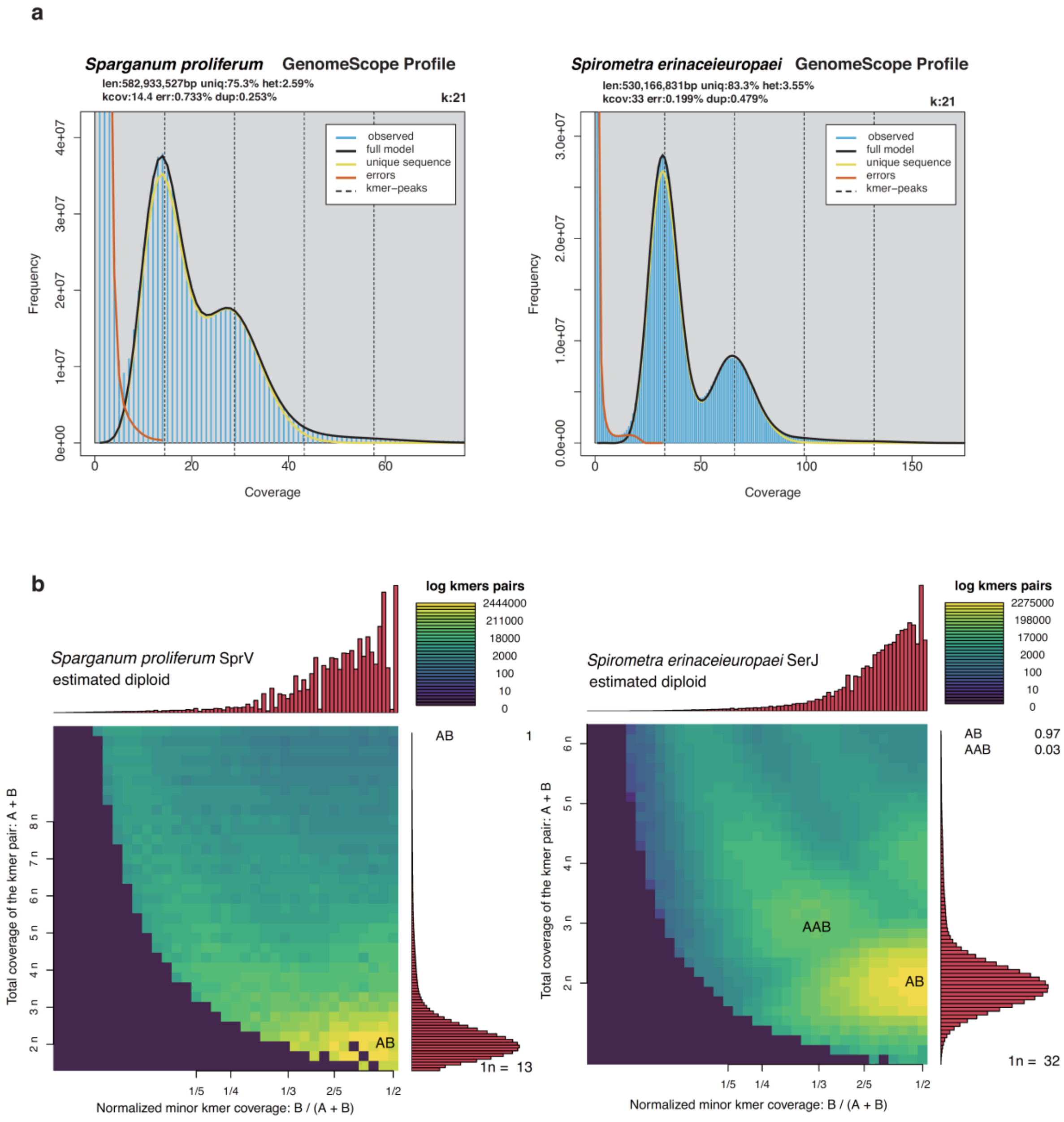

Supplementary Figure 1. (a) Histograms of the 23-mer depth distribution for *S. proliferum* and *S. erinaceieuropaei* were plotted by GenomeScope to estimate genome sizes, repeat contents, and heterozygosity levels. (b) Ploidy was estimated using Smudgeplots for *S. proliferum* and *S. erinaceieuropaei*.

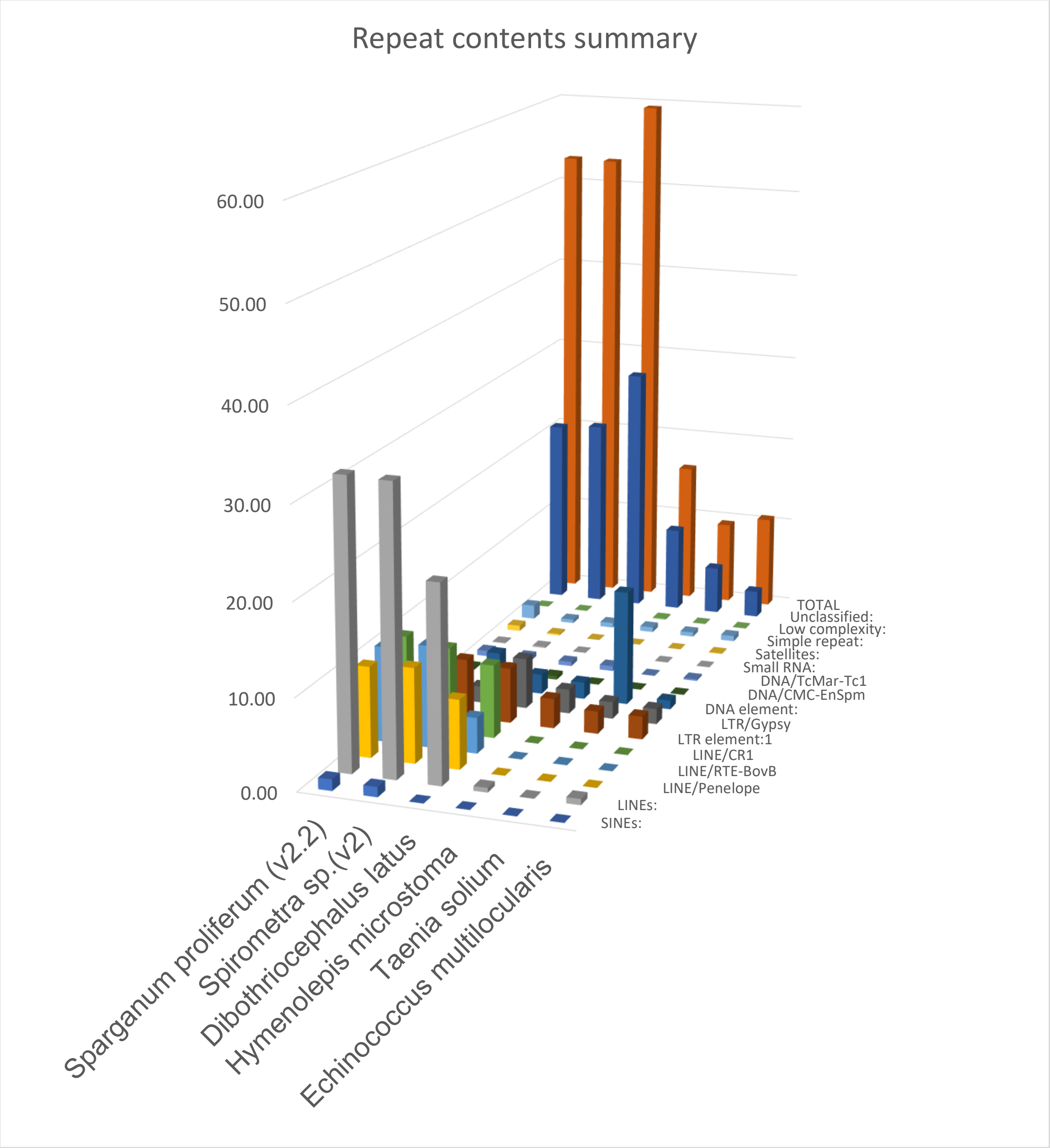

Supplementary Figure 2. Comparison of genome repeat contents among cestode species.

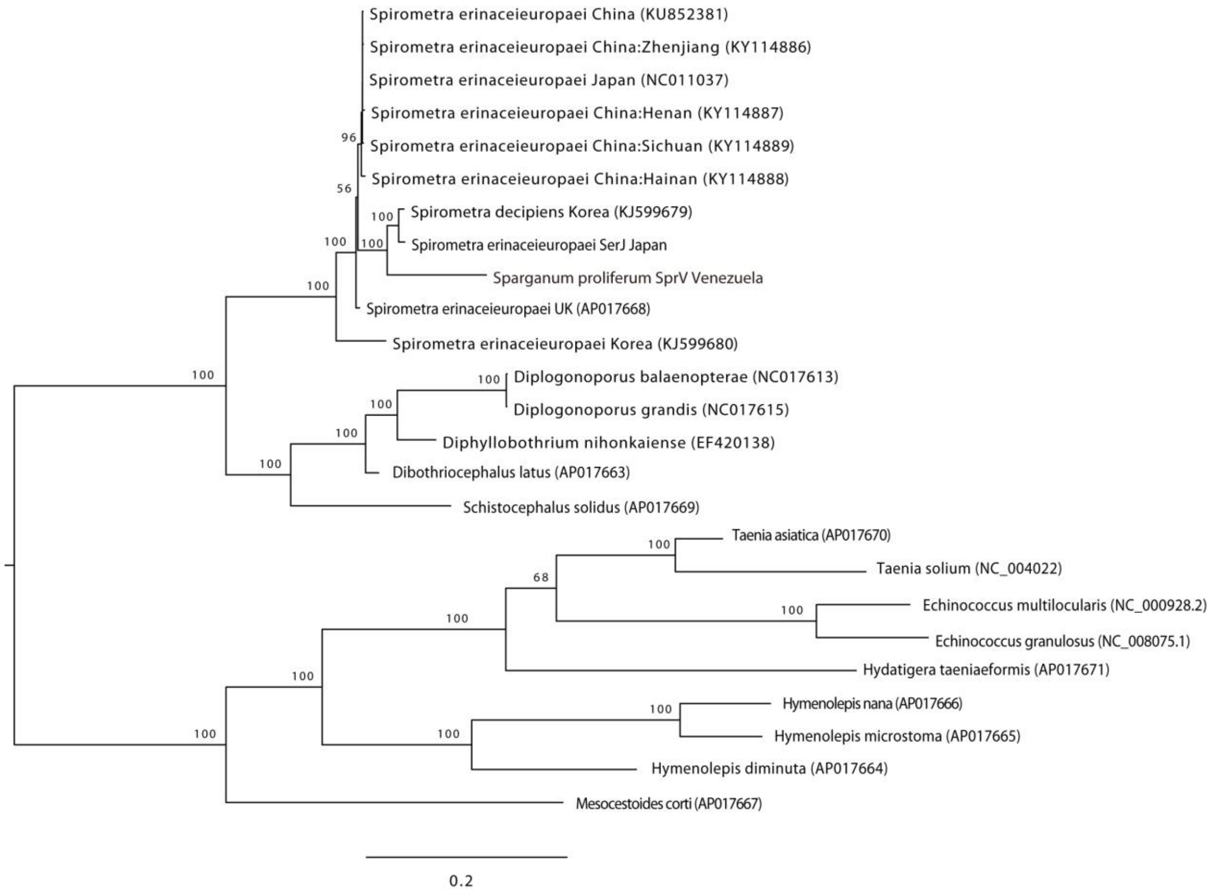

Supplementary Figure 3. Maximum-likelihood phylogenetic tree based on the mitochondrial genomes (12 protein-coding genes) of cestodes; amino acid sequences were aligned and phylogenetic analyses were performed with RAxML using the best-fitting empirical model of amino acid substitution with 1,000 bootstrap resampling replicates and the percentage support shown on the nodes. The scale bar shows the number of amino acid substitutions per site.

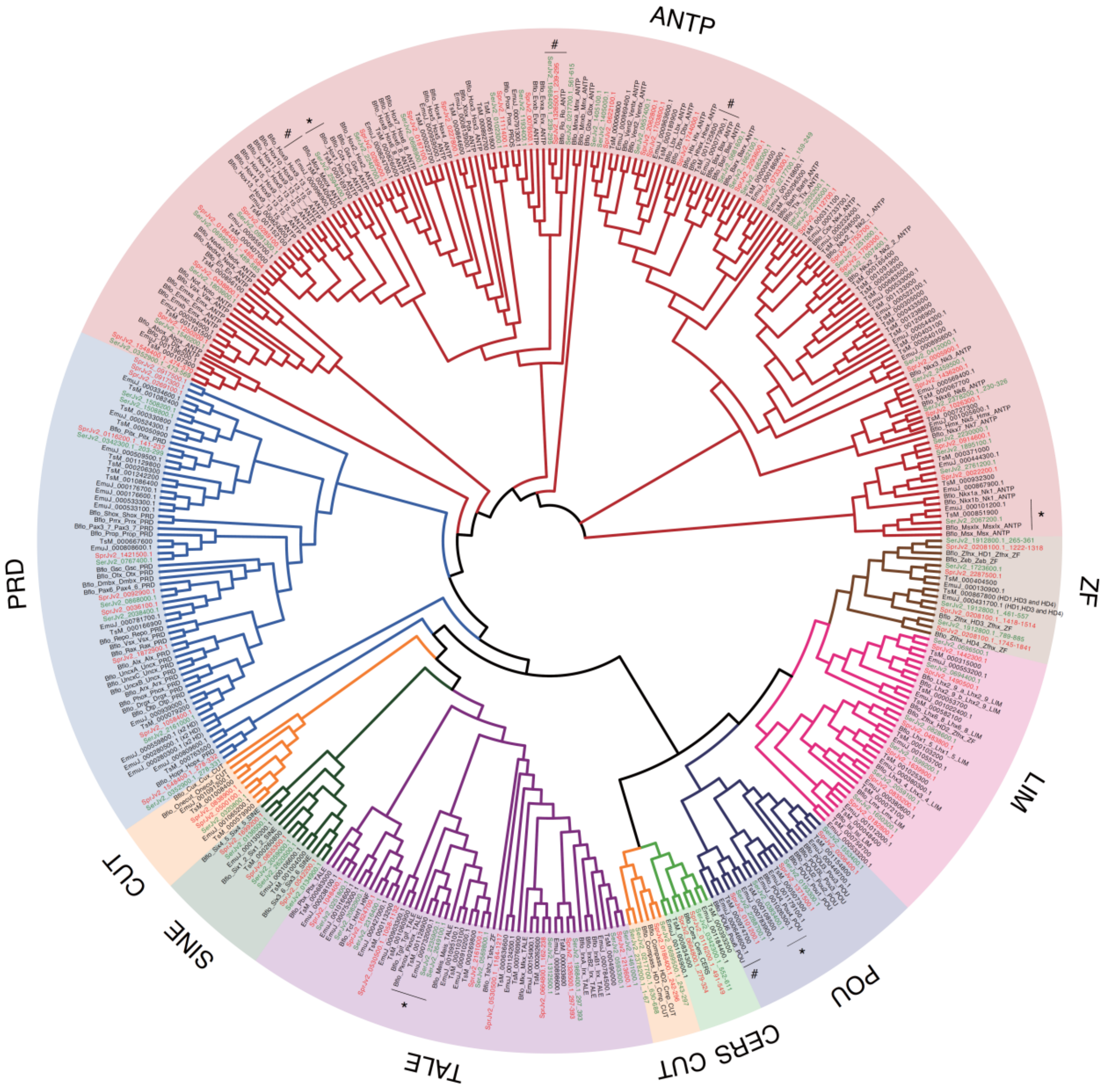

Supplementary Figure 4. Cladogram showing the diversity of homeobox genes in *S. proliferum* and *S. erinaceieuropaei* with the tapeworm species *T. solium* and *E. multilocularis* and the bilaterian *Branchiostoma floridae*.

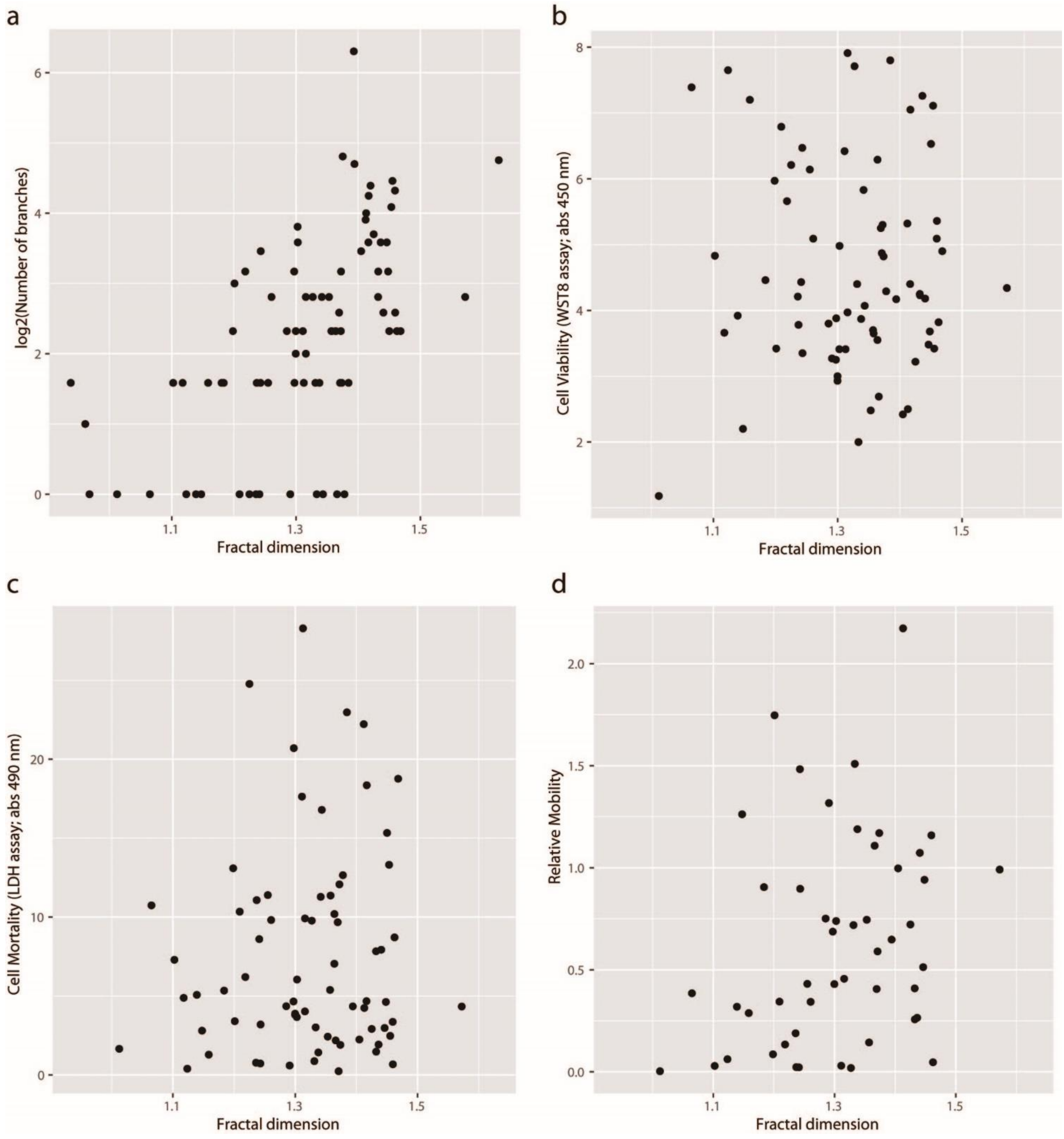

Supplementary Figure 5. Scatter plots showing relationships between morphological complexity value (fractal dimension) and a) number of branches, b) cell viability measurement using the WST-8 assay, c) cell mortality measurement using LDH assay, and d) mobility (relative average speed) of the plerocercoid worms.

a.

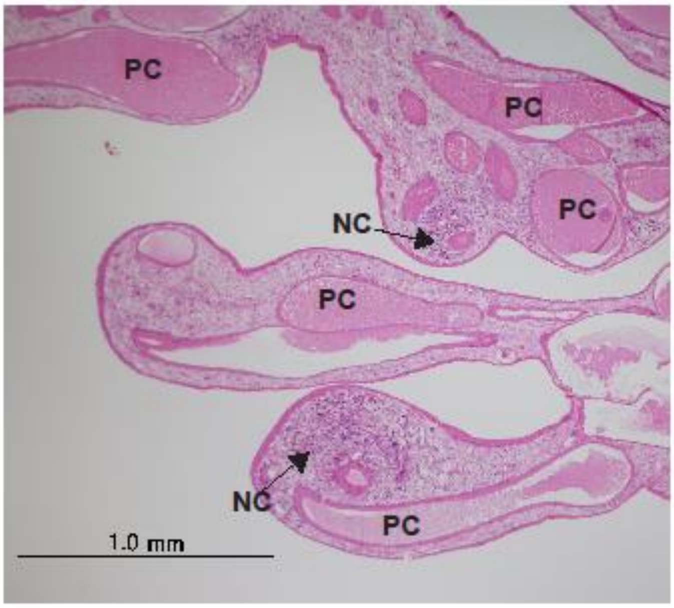

b.

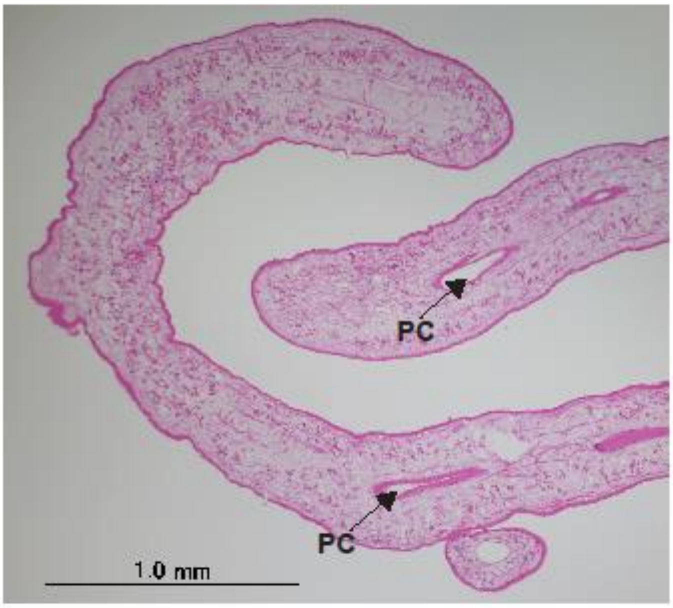

Supplementary Figure 6. Transversal histological cross-section of the Medusa-from (a) and the Wasabi-form (b) of *S. proliferum* plerocercoid stained with hematoxylin and eosin (H-E). The parenchymal cavity (PC) is highly developed in the medusa-form and the cavities were often filled with unidentified contents whereas they were empty in the wasabi-form. Concentrated nuclei (NC) were frequently observed in the Medusa-form.

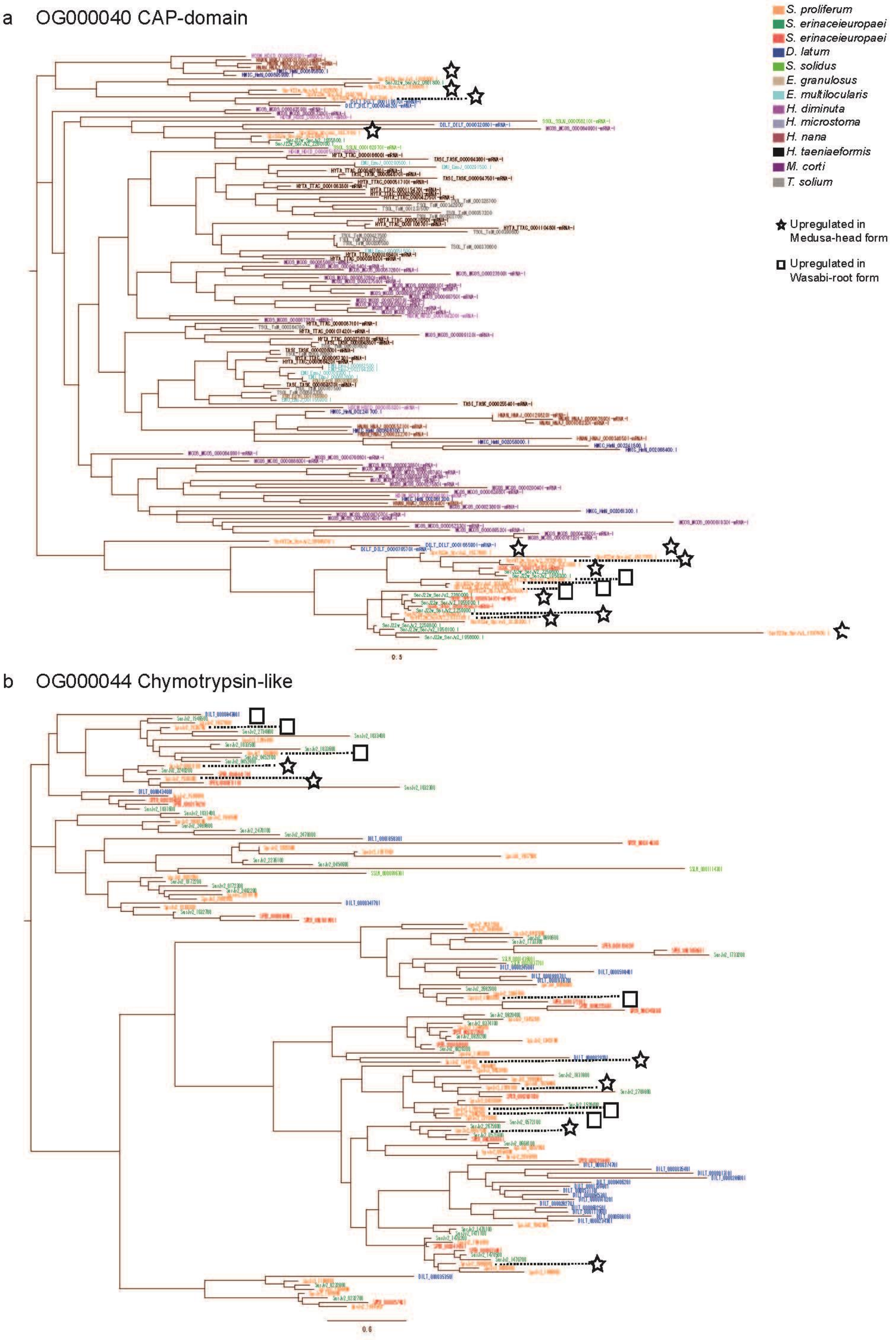
a OG000040 CAP-domain
b OG000044 Chymotrypsin-like
S. proliferum
S. erinaceieuropaei
S. erinaceieuropaei
D. latum
S. solidus
E. granulosus
E. multilocularis
H. diminuta
H. microstoma
H. nana
H. taeniaeformis
M. corti
T. solium
Upregulated in Medusa-head form
Upregulated in Wasabi-root form
0.5
0.6

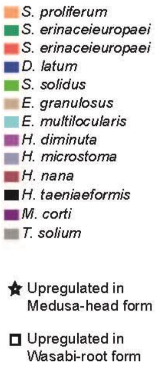

c OG000057 Fironectin type III

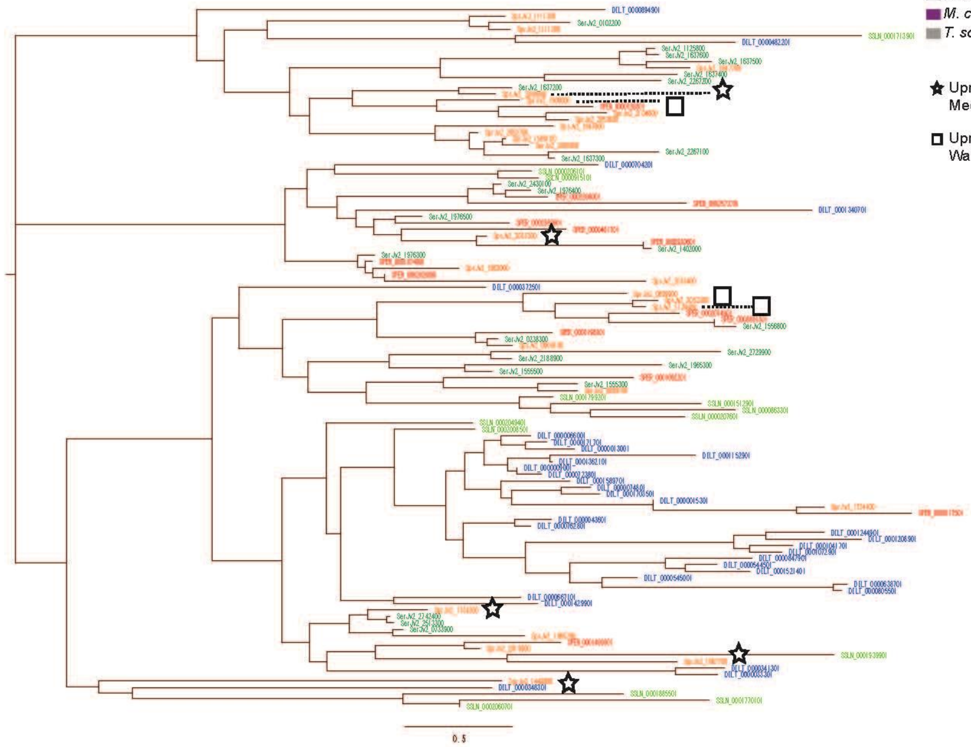

d OG000080 Leucyl aminopeptidase

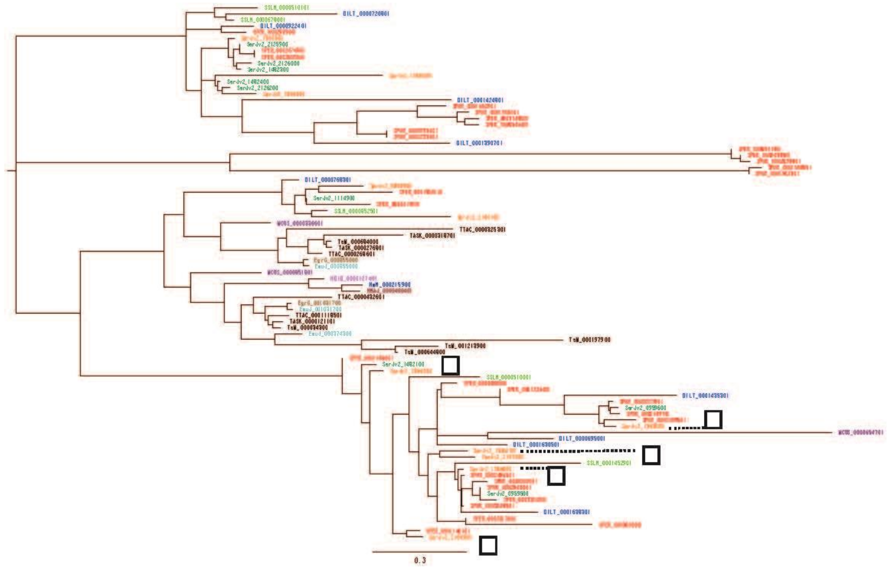

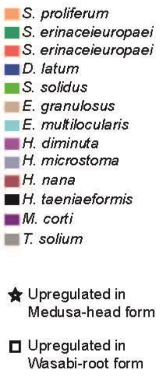

e OG000083 PISF

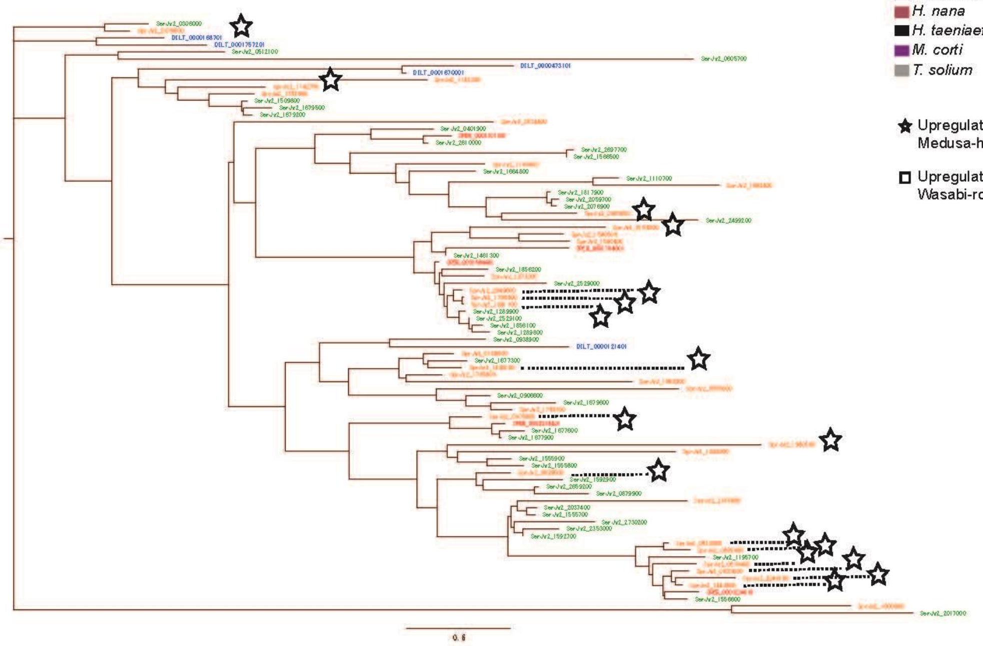

f OG000117 Tolloid-like

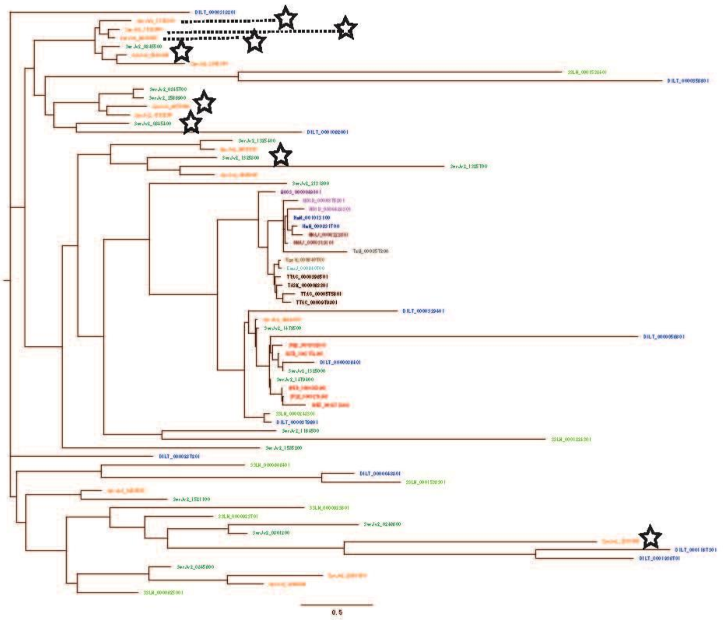

g OG000741 Tyrosine/ astacin

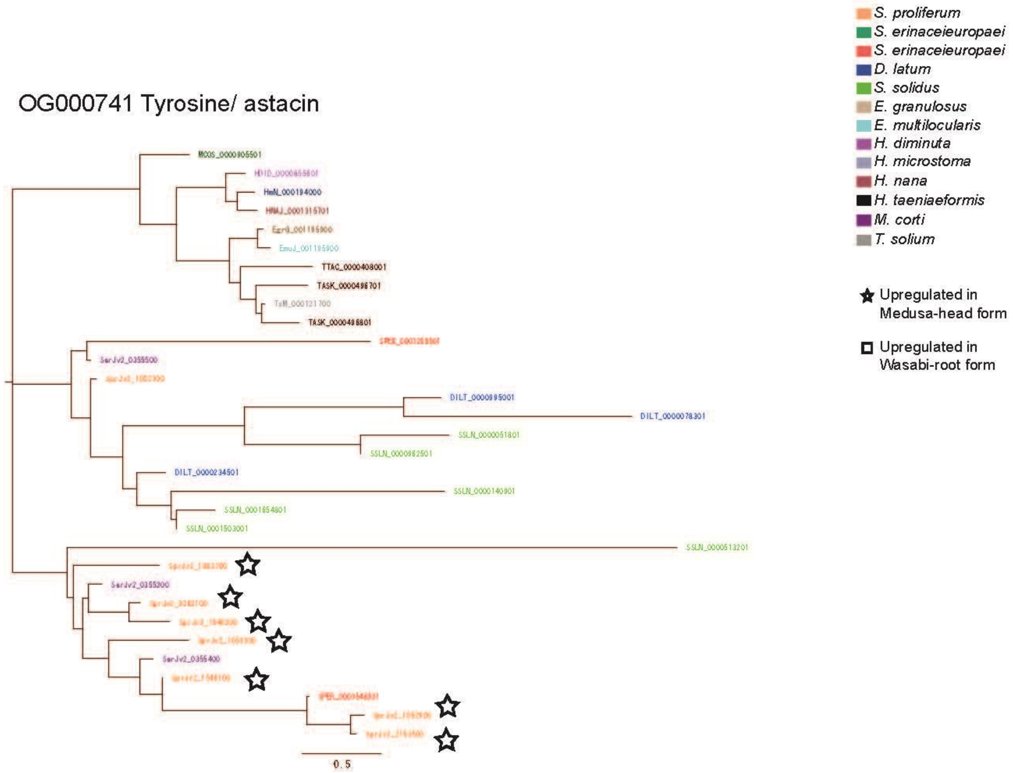

h OG000754 Tolloid-like

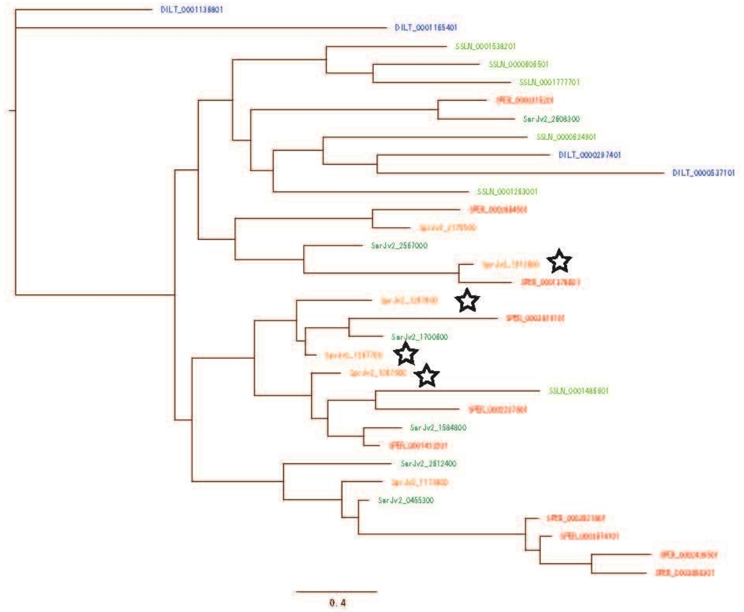

Supplementary Figure 7. Phylogenetic trees for nine orthofamilies that are differentially expressed between the medusa-head and wasabi-root forms of *S. proliferum*. Orthofamilies have been identified by Orthofinder analysis and include 12 species (*S. proliferum* = red, *S. erinaceieuropaei* =purple, *Dibothriocephalus latus* =blue, *Schistocephalus solidus* = green, *Hymenolepis diminuta* = magenta, *Hymenolepis nana* = dark red, *Hydatigera taeniaeformis* = black, *Taenia solium* = grey, *Echinococcus multilocularis* = cyan, *Echinococcus granulosus* = brown, *Mesocestoides corti* = dark green). The scale bar represents amino acid substitutions.

Supplementary Table 1. DNA and RNA sequencing libraries and data.

Species	Library name	Library type	Sample type	Accession number	Sequencing platform	Read length (bp)	Number of reads	Sequenced bases	Estimated insert size (bp)	Used for
**genomic DNA libraries used in assembly**										
Sparganum proliferum	lg2029noPCR	pair-end	plerocercoid	DRA009223	HiSeq 2500	101	51,258,062	5177M	377	Genome assembly
	lg2029_3kb	mate-pair	plerocercoid	DRA009223	HiSeq 2500	101	100,266,798	10127M	3215	Genome assembly
	lg2029_8kb	mate-pair	plerocercoid	DRA009223	HiSeq 2500	101	51,398,522	5191M	7836	Genome assembly
	pcrfree-Spro	pair-end	plerocercoid	DRA009223	MiSeq	301	44,229,096	13167M	584	Genome assembly
	mate_Spro	mate-pair	plerocercoid	DRA009223	MiSeq	301	22,149,640	6080M	312	Genome assembly
	600bSpro	pair-end	plerocercoid	DRA009223	HiSeq 2500	250	107,369,228	53684M	250	Genome assembly
	Spro_3k	mate-pair	plerocercoid	DRA009223	HiSeq 2500	150	376,992,404	28274M	2894	Genome assembly
	Spro_8k	mate-pair	plerocercoid	DRA009223	HiSeq 2500	150	189,733,668	28460M	7581	Genome assembly
	Spro_12k	mate-pair	plerocercoid	DRA009223	HiSeq 2500	150	183,357,312	27503M	11.8k	Genome assembly
	Spro_15k	mate-pair	plerocercoid	DRA009223	HiSeq 2500	150	182,115,962	27317M	13.8k	Genome assembly
Spirometra erinaceieuropaei	lg2109noPCR	pair-end	plerocercoid	DRA009224	HiSeq 2500	101	49,902,998	5040M	371	Genome assembly
	lg2109_3kb	mate-pair	plerocercoid	DRA009224	HiSeq 2500	101	90,244,528	9115M	3188	Genome assembly
	lg2109_8kb	mate-pair	plerocercoid	DRA009224	HiSeq 2500	101	49,300,478	4979M	7700	Genome assembly
	pcrfree-Ser	pair-end	plerocercoid	DRA009224	MiSeq	301	38,930,128	11600M	576	Genome assembly
	mate_Ser	mate-pair	plerocercoid	DRA009224	MiSeq	301	19,218,304	5344M	316	Genome assembly
	600bSer	pair-end	plerocercoid	DRA009224	HiSeq 2500	250	389,077,904	97269M	250	Genome assembly
	Spro_4k	mate-pair	plerocercoid	DRA009224	HiSeq 2500	150	121,195,426	18179M	2881	Genome assembly
	Spro_8k	mate-pair	plerocercoid	DRA009224	HiSeq 2500	150	124,835,432	18723M	8190	Genome assembly
	Spro_12k	mate-pair	plerocercoid	DRA009224	HiSeq 2500	150	117,647,898	17647M	12.8k	Genome assembly
	Spro_16k	mate-pair	plerocercoid	DRA009224	HiSeq 2500	150	114,111,590	17117M	15.6k	Genome assembly
**RNAseq libraries**										
Sparganum proliferum	lr731	polyA-stranded	plerocercoid	DRA009223	HiSeq 2500	101	46,654,052	4712M	160	Gene model prediction
	lr733	polyA-stranded	plerocercoid	DRA009223	HiSeq 2500	101	41,801,768	4222M	158	Gene model prediction
	Spro04L (W_2)	polyA-stranded	plerocercoid	DRA009223	HiSeq 2500	151	23,329,576	3499M	160	DE analysis
	Spro02L (W_1)	polyA-stranded	plerocercoid	DRA009223	HiSeq 2500	151	22,620,868	3393M	166	DE analysis
	Spro08H (M_0)	polyA-stranded	plerocercoid	DRA009223	HiSeq 2500	151	22,377,506	3357M	178	DE analysis
	Spro11H (M_1)	polyA-stranded	plerocercoid	DRA009223	HiSeq 2500	151	23,035,656	3455M	158	DE analysis
	Spro15H (M_5)	polyA-stranded	plerocercoid	DRA009223	HiSeq 2500	151	23,169,296	3475M	163	DE analysis
	Spro10L (W_3)	polyA-stranded	plerocercoid	DRA009223	HiSeq 2500	151	22,180,516	3327M	170	DE analysis
	Spro12H (M_2)	polyA-stranded	plerocercoid	DRA009223	HiSeq 2500	151	23,366,184	3505M	148	DE analysis
	Spro14H (M_4)	polyA-stranded	plerocercoid	DRA009223	HiSeq 2500	151	23,844,466	3577M	150	DE analysis
	Spro01L (W_0)	polyA-stranded	plerocercoid	DRA009223	HiSeq 2500	151	22,053,880	3308M	149	DE analysis
Spirometra erinaceieuropaei	lr734	polyA-stranded	plerocercoid	DRA009224	HiSeq 2500	101	42,195,312	4262M	167	Gene model prediction
	lr735	polyA-stranded	plerocercoid	DRA009224	HiSeq 2500	101	37,546,666	3792M	170	Gene model prediction

Supplementary Table 2. Pairwise genetic distance matrix of nuclear genes and mitochondrial genes among cestodes.

a. Genetic distance matrix of nuclear genes calculated from a concatenated aminoacid alignment of 205 single-copy genes

	DILT	EGU	EMU	HDIM	HMIC	HNAJ	HYTA	MCOS	SERE	SSOL	SerJ	SprV	TASI	TSOL
DILT														
EGU	0.366													
EMU	0.367	0.015												
HDIM	0.411	0.254	0.256											
HMIC	0.417	0.264	0.266	0.092										
HNAJ	0.416	0.265	0.266	0.098	0.055									
HYTA	0.369	0.091	0.094	0.257	0.268	0.269								
MCOS	0.343	0.219	0.221	0.293	0.301	0.301	0.225							
SERE	0.096	0.369	0.370	0.414	0.420	0.420	0.372	0.344						
SSOL	0.084	0.365	0.365	0.411	0.416	0.415	0.367	0.341	0.083					
SerJ	0.090	0.360	0.361	0.405	0.411	0.411	0.363	0.335	0.016	0.075				
SprV	0.092	0.363	0.364	0.408	0.415	0.415	0.366	0.338	0.033	0.077	0.022			
TASI	0.364	0.076	0.080	0.252	0.263	0.263	0.078	0.220	0.367	0.363	0.358	0.361		
TSOL	0.365	0.076	0.079	0.252	0.263	0.263	0.077	0.220	0.368	0.363	0.359	0.362	0.018	

DILT: Dibothriocephalus latus, EGU: Echinococcus granulosus, EMU: Echinococcus multilocularis, HDIM: Hymenolepis diminuta, HMIC: Hymenolepis microstoma, HNAN: Hymenolepis nana, HYTA: Hydatigera taeniaeformis, MCOS: Mesocestoides corti, SERE: Spirometra erinaceieuropaei UK, SSOL: Schistocephalus solidus, SerJ: Spirometra erinaceieuropaei Japan, SprV: Sparganum proliferum, TASI: Taenia asiatica, TSOL: Taenia solium

b. Genetic distance matrix of mitochndrial genes calculated from a concatenated aminoacid alignment of 12 genes

	D_balaen	D_grandi	DILT	D_nihonka	EGU	EMU	HDID	HMIC	HNAJ	MCOS	S_decipien	SPER	NC01103	SSLN	TASK	TSOL	HYTA	SerJ	SprV	KJ59968	KU85238	KY11488	KY11488	KY11488	KY11488
D_balaenop																									
D_grandis	0.001																								
DILT	0.113	0.113																							
D_nihonkai	0.110	0.110	0.066																						
EGU	0.475	0.475	0.458	0.472																					
EMU	0.476	0.475	0.456	0.471	0.142																				
HDID	0.422	0.421	0.392	0.424	0.390	0.387																			
HMIC	0.450	0.449	0.421	0.449	0.420	0.426	0.264																		
HNAJ	0.463	0.462	0.425	0.455	0.425	0.425	0.271	0.134																	
MCOS	0.420	0.420	0.394	0.422	0.405	0.405	0.359	0.375	0.385																
S_decipien	0.216	0.215	0.202	0.210	0.467	0.464	0.431	0.443	0.452	0.412															
SPER	0.209	0.209	0.171	0.200	0.454	0.452	0.401	0.415	0.422	0.384	0.042														
NC011037	0.209	0.209	0.172	0.200	0.454	0.453	0.401	0.416	0.424	0.385	0.036	0.009													
SSLN	0.197	0.196	0.164	0.195	0.473	0.478	0.403	0.429	0.427	0.404	0.234	0.203	0.202												
TASK	0.447	0.447	0.411	0.444	0.300	0.291	0.337	0.376	0.378	0.366	0.448	0.416	0.420	0.430											
TSOL	0.467	0.467	0.460	0.465	0.332	0.322	0.398	0.420	0.427	0.417	0.459	0.460	0.459	0.471	0.173										
HYTA	0.490	0.490	0.458	0.494	0.354	0.356	0.388	0.412	0.425	0.394	0.489	0.457	0.458	0.454	0.303	0.359									
SerJ	0.214	0.213	0.201	0.210	0.463	0.461	0.430	0.442	0.451	0.411	0.010	0.038	0.042	0.233	0.445	0.459	0.487								
SprV	0.211	0.211	0.205	0.208	0.466	0.464	0.424	0.442	0.449	0.412	0.085	0.099	0.100	0.226	0.441	0.455	0.486	0.085							
KJ599680	0.218	0.217	0.183	0.208	0.460	0.459	0.400	0.417	0.432	0.389	0.088	0.057	0.058	0.213	0.423	0.460	0.461	0.086	0.108						
KU852381	0.210	0.209	0.172	0.200	0.454	0.454	0.401	0.416	0.424	0.385	0.036	0.009	0.000	0.203	0.420	0.460	0.459	0.042	0.100	0.059					
KY114886	0.210	0.209	0.172	0.200	0.453	0.454	0.401	0.416	0.424	0.385	0.037	0.009	0.001	0.203	0.420	0.460	0.458	0.042	0.101	0.058	0.001				
KY114887	0.211	0.210	0.173	0.201	0.455	0.455	0.403	0.417	0.425	0.387	0.038	0.010	0.002	0.204	0.422	0.461	0.460	0.043	0.102	0.060	0.002	0.002			
KY114888	0.212	0.211	0.175	0.203	0.459	0.456	0.404	0.419	0.428	0.389	0.040	0.011	0.004	0.206	0.422	0.464	0.463	0.044	0.102	0.061	0.005	0.005	0.006		
KY114889	0.210	0.209	0.173	0.200	0.455	0.455	0.402	0.417	0.425	0.385	0.037	0.010	0.001	0.203	0.421	0.460	0.459	0.043	0.101	0.060	0.001	0.002	0.003	0.005	

D_balaenop: Diplogonoporus balaenopterae (NC017613), D_grandis: Diplogonoporus grandis (NC017615), DILT: Dibothriocephalus latus (AP017663), D_nihonkai: Diphyllobothrium nihonkaiense (EF420138), EGU: Echinococcus granulosus (NC_008075.1), EMU: Echinococcus multilocularis (NC_000928.2), HDID: Hymenolepis diminuta (AP017664), HMIC: Hymenolepis microstoma (AP017665), HNAJ: Hymenolepis nana (AP017666), MCOS: Mesocestoides corti (AP017667), S_decipien: Spirometra decipiens Korea (KJ599679), SERE: Spirometra erinaceieuropaei UK (AP017668), NC011037: Spirometra erinaceieuropaei Japan (NC011037), SSLN: Schistocephalus solidus (AP017669), TASK: Taenia asiatica (AP017670), TSOL: Taenia solium (NC_004022), TTAC: Hydatigera taeniaeformis (AP017671), SerJ: Spirometra erinaceieuropaei SerJ, SprV: Sparganum proliferum SprV, KJ599680: Spirometra erinaceieuropaei Korea, KU852381: Spirometra erinaceieuropaei China, KY114886: Spirometra erinaceieuropaei China, KY114887: Spirometra erinaceieuropaei China, KY114888: Spirometra erinaceieuropaei China, KY114889: Spirometra erinaceieuropaei China

Supplementary Table 3. Estimation of gene family expansion and contraction using CAFE.

	Families Expansions	Families Equal	Families Contractions	Families Lost	Genes Gained	Genes Lost	Average Expansion	Siginicant Expansions	Significant Contractions	Total Significant Changes
Spirometra erinaceieuropaei	1406	10655	1115	829	1823	1155	0.050698	35	8	43
Sparganum proliferum	554	10808	1814	1172	752	1904	-0.087432	17	14	29
<1>	276	10563	2337	1673	463	2456	-0.15126	17	3	20
Schistocephalus solidus	726	10464	1986	1482	1200	2142	-0.071494	20	14	34
Dibothriocephalus latus	2246	9632	1298	1148	3689	1438	0.170841	50	7	57
<5>	38	11507	1631	1618	38	1635	-0.121205	0	1	1
<3>	481	9891	2804	2637	728	2833	-0.15976	5	0	5
Echinococcus multilocularis	402	12101	673	511	607	692	-0.006451	13	3	16
Taenia asiatica	432	11276	1468	1315	703	1497	-0.060261	21	7	28
<9>	111	12237	828	742	175	837	-0.050243	8	1	9
Hymenolepis microstoma	859	10091	2226	2017	1459	2267	-0.061324	17	4	21
<11>	21	12647	508	428	29	519	-0.037189	1	1	2
Mesocestoides corti	586	9427	3163	2934	888	3220	-0.176988	8	2	10
<13>	35	7792	5349	4022	53	5429	-0.408015	0	3	3

Labeled tree:
(((Spirometra_erinaceieuropaei,Sparganum_proliferum)<1>,(Schistocephalus_solidus,Dibothriocephalus_latus)<5>)<3>,(((Echinococcus_multilocularis,Taenia_asiatica)<9>,Hymenolepis_microstoma)<11>,Mesocestoides_corti)<13>)

Supplementary Table 4. Positively selected genes in the S. proliferum lineage that was identified using dN/dS branch-site model.

OrthoGroup	Gene ID	Description	pfam domains	GO terms	Alt_lnl	Nul_lnl	LRT	p-value	lfdr
OG0003210	SprJv2_0424600.1	Similar to MPP6 MAGUK p55Subfamily member 6 (HomoSapiens OX=9606)	Guanylate_kin, PDZ, SH3_2	GO:0005515	-2028.361422	-2041.226	25.729156	3.92847E-07	4.98E-06
OG0003459	SprJv2_1968500.1	Similar to PARD3 Partitioning defective 3 homolog (HomoSapiens OX=9606)	PDZ	GO:0005515, GO:0044464, GO:0044763	-697.637	-705.279	15.284	9.24967E-05	0.002967486
OG0004165	SprJv2_1249400.1	Similar to Dars Aspartate--tRNA ligase, cytoplasmic (Mus musculus OX=10090)	tRNA_anti-codon, tRNA-synt_2d, tRNA-synt_2	GO:0003676, GO:0004815, GO:0005524, GO:0005737, GO:0006422	-8089.365	-8102.57	26.41	2.76107E-07	3.27E-06
OG0004294	SprJv2_0255600.1	Similar to DNAJC9 DnaJ homologSubfamily C member 9 (HomoSapiens OX=9606)	DnaJ	NA	-3852.466	-3862.934	20.936	4.74886E-06	9.40E-05
OG0004331	SprJv2_0084300.1	Similar to RPIA Ribose-5-phosphate isomerase (Fragment) (Bos taurus OX=9913)	Rib_5-P_isom_A	GO:0004751, GO:0009052	-1113.747	-1126.034	24.574	7.15101E-07	1.02E-05
OG0004431	SprJv2_0179300.1	Similar to Dcaf7 DDB1- and CUL4-associated factor 7 (Mus musculus OX=10090)	WD40	GO:0003824, GO:0005515, GO:0016021	-1540.98	-1551.472	20.984	4.63135E-06	9.12E-05
OG0004624	SprJv2_0115100.1	Similar to Eif4g3 Eukaryotic translation initiation factor 4 gamma 3 (Mus musculus OX=10090)	MA3, MIF4G	GO:0003723, GO:0005515	-11121.011	-11133.742	25.462	4.51183E-07	5.87E-06
OG0004635	SprJv2_1750500.1	Similar to Irf4 Interferon regulatory factor 4 (Mus musculus OX=10090)	IRF-3, IRF	GO:0000975, GO:0003700, GO:0005515, GO:0006355	-7792.092	-7841.809	99.434	2.02811E-23	9.50E-08
OG0004652	SprJv2_1450100.1	Similar to PMM2 Phosphomannomutase 2 (Macaca fascicularis OX=9541)	Hydrolase_3, PMM	GO:0004615, GO:0005737, GO:0009298	-3328.641	-3358.719	60.156	8.76294E-15	9.50E-08
OG0004687	SprJv2_0170500.1	Similar to AdenylosuccinateSynthetase (Schistosoma japonicum OX=6182)	Adenylsucc_synt	GO:0004019, GO:0005525, GO:0006167, GO:0046872	-3264.47	-3281.408	33.876	5.87388E-09	9.50E-08
OG0004774	SprJv2_1752700.1	Similar to RPL21 60S ribosomal protein L21 (SusScrofa OX=9823)	Ribosomal_L21e	GO:0003735, GO:0006412, GO:0022625	-2284.732	-2319.624	69.784	6.61674E-17	9.50E-08
OG0004810	SprJv2_1089200.1	Similar to Dynein intermediate chain 2, ciliary (Heliocidaris crassispina OX=1043166)	WD40	GO:0005515	-3944.697	-3960.062	30.73	2.96545E-08	2.68E-07
OG0004816	SprJv2_0954800.1	Similar toSnu13 NHP2-like protein 1 (Xenopus tropicalis OX=8364)	Ribosomal_L7Ae	GO:0003723, GO:0005525, GO:0005730, GO:0007264, GO:0030529, GO:0042254	-866.625	-882.501	31.752	1.75169E-08	1.59E-07
OG0004930	SprJv2_1925800.1	Similar to Chmp5 Charged multivesicular body protein 5 (Rattus norvegicus OX=10116)	Snf7	GO:0007034	-3934.295	-3970.043	71.496	2.77821E-17	9.50E-08
OG0004959	SprJv2_0988800.1	Similar to rbm18 Probable RNA-binding protein 18 (Danio rerio OX=7955)	RRM_1	GO:0000166, GO:0003676	-2862.945	-2874.245	22.6	1.99485E-06	3.41E-05
OG0004995	SprJv2_1462800.1	Similar to Wbp4 WW domain-binding protein 4 (Mus musculus OX=10090)	WW, zf-U1	GO:0003676, GO:0005515, GO:0005634, GO:0008270	-4414.4	-4422.681	16.562	4.70851E-05	0.001347423
OG0005044	SprJv2_0122900.1	Similar to Apex1 DNA-(apurinic or apyrimidinicSite) lyase (Rattus norvegicus OX=10116)	Exo_endo_phos	GO:0003677, GO:0003906, GO:0005622, GO:0006281, GO:0046872, GO:0090305	-2521.644	-2535.397	27.506	1.56608E-07	1.68E-06
OG0005221	SprJv2_0845000.1	Similar to Polr2d DNA-directed RNA polymerase IISubunit RPB4 (Mus musculus OX=10090)	NA	EC:2.7.7.6, GO:0000166, GO:0000288, GO:0000932, GO:0003697, GO:0003727, GO:0003899, GO:0005665, GO:0006367, GO:0031369, GO:0031990, GO:0034402, GO:0045948	-1439.122	-1461.359	44.474	2.57758E-11	9.50E-08
OG0005228	SprJv2_1289900.1	Similar to Hnrnpl Heterogeneous nuclear ribonucleoprotein L (Rattus norvegicus OX=10116)	Limkain-b1, RRM_5, RRM_occluded, RRM_1	GO:0000166, GO:0003676	-3303.651	-3349.823	92.344	7.28465E-22	9.50E-08
OG0005244	SprJv2_0573200.1	Similar to TOP1 DNA topoisomerase 1 (HomoSapiens OX=9606)	Topo_C_assoc, Topoisom_I_N, Topoisom_I	GO:0003677, GO:0003917, GO:0003918, GO:0005694, GO:0006265	-6424.146	-6475.501	102.71	3.87981E-24	9.50E-08
OG0005375	SprJv2_1665400.1	Similar to Ptbp1 Polypyrimidine tract-binding protein 1 (Mus musculus OX=10090)	RRM_5, RRM_1	GO:0000166, GO:0003676	-6654.573	-6740.483	171.82	2.9626E-39	9.50E-08
OG0005397	SprJv2_1383000.1	Similar to Pdss1 Decaprenyl-diphosphateSynthaseSubunit 1 (Mus musculus OX=10090)	polyprenyl_synt	GO:0008299	-4245.378	-4255.493	20.23	6.86676E-06	0.000144509
OG0005425	SprJv2_0587300.1	Protein of unknown function	Usp	GO:0006950	-2599.23	-2614.183	29.906	4.53507E-08	4.17E-07
OG0005451	SprJv2_1003400.1	Similar to UQCRFS1 Cytochrome b-c1 complexSubunit Rieske, mitochondrial (SaimiriSciureus OX=9521)	Rieske, UCR_TM	GO:0005743, GO:0008121, GO:0046872, GO:0051537, GO:0055114, GO:0070469, GO:1902600	-3212.191	-3224.023	23.664	1.14708E-06	1.78E-05
OG0005478	SprJv2_1872300.1	Similar to eef1aknmt eEF1A lysine and N-terminal methyltransferase (Danio rerio OX=7955)	Methyltransf_25, Methyltransf_31, MTS	NA	-7054.077	-7094.959	81.764	1.53346E-19	9.50E-08
OG0005519	SprJv2_1546800.1	Similar to HIKESHI Protein Hikeshi (Gallus gallus OX=9031)	DUF775	NA	-2576.984	-2593.502	33.036	9.04681E-09	9.50E-08
OG0005556	SprJv2_0421300.1	Similar toSan Probable N-acetyltransferaseSan (Drosophila melanogaster OX=7227)	NA	GO:0004596, GO:0006474, GO:0031248	-1802.837	-1831.881	58.088	2.50651E-14	9.50E-08
OG0005680	SprJv2_1190800.1	Similar to Ak6 Adenylate kinase isoenzyme 6 (Mus musculus OX=10090)	AAA_18	EC:2.7.4.3, EC:3.6.1.3, GO:0004017, GO:0005524, GO:0005634, GO:0016887, GO:0046939	-2416.885	-2449.08	64.39	1.02075E-15	9.50E-08
OG0005709	SprJv2_0797100.1	Similar to ELF5A-3 Eukaryotic translation initiation factor 5A-3 (Arabidopsis thaliana OX=3702)	eIF-5a	GO:0003743, GO:0003746, GO:0006413, GO:0006452, GO:0043022, GO:0045901, GO:0045905	-1170.176	-1185.079	29.806	4.77512E-08	4.41E-07
OG0005844	SprJv2_0177500.1	Similar to Probable Ras-related protein Rab-4A (Echinococcus multilocularis OX=6211)	Arf, Ras, Roc	GO:0005525, GO:0005768, GO:0005886, GO:0007264, GO:0015031, GO:0030100	-2000.402	-2026.204	51.604	6.79031E-13	9.50E-08
OG0005872	SprJv2_2253800.1	Similar to HGV2 Protein HGV2 (Halocynthia roretzi OX=7729)	SHNi-TPR	NA	-1142.844	-1162.733	39.778	2.84532E-10	9.50E-08
OG0005884	SprJv2_0722200.1	Similar to DERL2 Derlin-2 (Pongo abelii OX=9601)	DER1	GO:0005789, GO:0016021	-2052.789	-2058.726	11.874	0.00056923	0.031606777
OG0005940	SprJv2_2067800.1	Similar to BSG Basigin (Fragment) (Cricetulus griseus OX=10029)	Ig_3, ig, I-set	GO:0005515, GO:0016020	-3109.873	-3117.712	15.678	7.50926E-05	0.002320232
OG0005951	SprJv2_0442300.1	Similar to Toll-6 Toll-like receptor 6 (Drosophila melanogaster OX=7227)	LRR_4, LRR_5, LRR_6, LRR_8, LRR_1	GO:0005515	-10224.802	-10240.529	31.454	2.04221E-08	1.84E-07
OG0006108	SprJv2_1564800.1	Similar to F58A4.6 Uncharacterized protein F58A4.6 (Caenorhabditis elegans OX=6239)	DUF4807	NA	-1380.055	-1405.413	50.716	1.06746E-12	9.50E-08

lnL Model null: log likelihood for null model with fixed dN/dS ratios.

lnL Model alt: log likelihood for alternative model which allows having unfixed dN/dS values at the branch.

LRT: 2(lnL Model alt - lnL Model null) for the likelihood ratio test .

P values are from LRT and adjusted for multiple testing (lfdr).

Supplementary Table 5. Genes under relaxed selection in the *S. proliferum* lineage detected by the RELAX tool.

OrtFam	Gene ID	Description	pFAM	GO term	Relaxation parameter (K)	Raw p-value	Adjusted p-value (FDR)
OG0003551	SprJv2_0299200.1	Similar to PCDHGC4 Protocadherin gamma-C4 (Pan troglodytes OX=9598)	Cadherin_3, Cadherin	GO:0005509, GO:0005886, GO:0007156	0.32	0.0305	0.0351
OG0003688	SprJv2_0281900.1	Similar to PCDHA1 Protocadherin alpha-1 (HomoSapiens OX=9606)	Cadherin_2, Cadherin	GO:0005509, GO:0005886, GO:0007156	0	0.0003	0.0009
OG0004228	SprJv2_0016500.1	Similar to NDUFA8 NADH dehydrogenase [ubiquinone] 1 alphaSubcomplexSubunit 8 (Pan troglodytes OX=9598)	CX9C	GO:0005743, GO:0016491, GO:0055114, GO:0070469	0	0.0047	0.0088
OG0004437	SprJv2_0330000.1	Similar to KDR Vascular endothelial growth factor receptor 2 (HomoSapiens OX=9606)	Pkinase, Pkinase_Tyr	GO:0004713, GO:0005515, GO:0005524, GO:0006468	0.33	0.0274	0.0329
OG0004566	SprJv2_0720900.1	Protein of unknown function	NA	NA	0	0.0045	0.0088
OG0004917	SprJv2_0086200.1	Similar to ROBO2 Roundabout homolog 2 (HomoSapiens OX=9606)	Ig_2, Ig_3, fn3, ig, I-set, V-set	GO:0005515	0	0.0001	0.0003
OG0005389	SprJv2_1653800.1	Similar toSlit3Slit homolog 3 protein (Rattus norvegicus OX=10116)	LRR_5, LRR_8	GO:0005515	0.18	0.0001	0.0003
OG0005473	SprJv2_0826200.1	Similar to ARHGAP21 Rho GTPase-activating protein 21 (HomoSapiens OX=9606)	RhoGAP	GO:0007165	0.39	0.0002	0.0006
OG0005835	SprJv2_0384300.1	Similar to dla Delta-like protein A (Danio rerio OX=7955)	hEGF, EGF	GO:0005509, GO:0005515	0.18	0.0257	0.0329
OG0005998	SprJv2_0370900.1	Similar to TMX1 Thioredoxin-related transmembrane protein 1 (HomoSapiens OX=9606)	Thioredoxin	GO:0016020, GO:0045454	0.06	0.03	0.0354

Supplementary Table 6. Morphology, viability and mobility of *S. proliferum* worms isolated from infected mice.

Worm ID	Mouse ID	Number of branches	Nimber of junctions	Number of triple points	Number of quadruple points	Fractal dimension	Worm size (area)	WST8 abs.450nm	LDH abs.490nm	adjusted WST8	adjusted LDH	Mobility (relative average speed)
M0	2018.1	9	4	4	0	1.3725	3.65	ND	ND	ND	ND	ND
M1	2018.2	79	44	37	4	1.393	27.96	ND	ND	ND	ND	ND
M2	2018.3	21	12	12	0	1.4199	7.59	ND	ND	ND	ND	ND
M3	2018.4	27	14	12	2	1.6262	5.14	ND	ND	ND	ND	ND
M4	2018.5	28	15	13	2	1.3752	6.9	ND	ND	ND	ND	ND
W0	2018.6	3	1	1	0	1.1803	2.04	ND	ND	ND	ND	ND
W1	2018.2	3	1	1	0	0.9377	1.17	ND	ND	ND	ND	ND
W2	2018.7	1	0	0	0	0.9678	1.49	ND	ND	ND	ND	ND
W3	2018.8	2	1	0	0	0.961	0.68	ND	ND	ND	ND	ND
P1A01	2020.1-3	20	10	10	0	1.4593	9.348	0.476	0.314	5.09	3.36	ND
P1A02	2020.1-3	17	7	5	1	1.4534	3.757	0.267	0.5	7.11	13.31	ND
P1A03	2020.1-3	22	10	7	2	1.4552	9.276	0.317	0.229	3.42	2.47	ND
P1A04	2020.1-3	5	2	2	0	1.364	4.927	0.31	0.502	6.29	10.19	ND
P1A05	2020.1-3	12	6	5	0	1.4165	5.821	0.256	0.272	4.4	4.67	ND
P1A06	2020.1-3	5	2	2	0	1.4501	2.113	0.138	0.324	6.53	15.33	ND
P1B01	2020.1-3	7	3	2	1	1.3416	2.917	0.17	0.329	5.83	11.28	ND
P1B02	2020.1-3	5	2	2	0	1.3722	2.735	0.145	0.33	5.3	12.07	ND
P1B03	2020.1-3	12	5	4	0	1.3029	3.775	0.188	0.228	4.98	6.04	ND
P1B04	2020.1-3	1	0	0	0	1.3779	1.423	0.061	0.18	4.29	12.65	ND
P1B05	2020.1-3	4	1	0	1	1.3159	2.604	0.206	0.258	7.91	9.91	ND
P1B06	2020.1-3	5	2	2	0	1.3577	2.465	0.09	0.28	3.65	11.36	ND
P1C01	2020.1-3	3	1	1	0	1.3845	2.116	0.165	0.486	7.8	22.97	ND
P1C02	2020.1-3	15	8	8	0	1.412	5.414	0.288	1.203	5.32	22.22	ND
P1C03	2020.1-3	5	3	3	0	1.4682	3.572	0.175	0.67	4.9	18.76	ND
P1C04	2020.1-3	19	10	9	1	1.4169	5.149	0.363	0.945	7.05	18.35	ND
P1C05	2020.1-3	5	2	2	0	1.3639	3.609	0.128	0.254	3.55	7.04	ND
P1D01	2020.1-3	1	0	0	0	1.2251	3.222	0.2	0.798	6.21	24.77	ND
P1D02	2020.1-3	3	1	1	0	1.1176	2.625	0.096	0.128	3.66	4.88	ND
P1D03	2020.1-3	3	1	1	0	1.3126	7.648	0.261	2.164	3.41	28.29	ND
P1D04	2020.1-3	3	1	1	0	1.2976	2.372	0.092	0.491	3.88	20.7	ND
P1D05	2020.1-3	4	1	0	1	1.2996	3.042	0.089	0.116	2.93	3.81	ND
P1D06	2020.1-3	1	0	0	0	1.3434	0.983	0.04	0.165	4.07	16.79	ND
P2A02	2020.1-3	12	5	4	1	1.4362	2.397	0.174	0.046	7.26	1.92	0.265
P2A03	2020.1-3	9	4	4	0	1.4323	2.319	0.098	0.034	4.23	1.47	0.257
P2A04	2020.1-3	3	1	1	0	1.3737	2.636	0.127	0.05	4.82	1.9	1.17
P2A05	2020.1-3	6	2	1	1	1.4596	3.734	0.2	0.025	5.36	0.67	1.159
P2A06	2020.1-3	3	1	1	0	1.2429	1.252	0.081	0.009	6.47	0.72	1.483
P2B01	2020.1-3	3	1	1	0	1.3377	2.12	0.082	0.03	3.87	1.42	1.189
P2B02	2020.1-3	5	2	2	0	1.2997	2.266	0.068	0.088	3	3.88	0.43
P2B03	2020.1-3	3	1	1	0	1.1586	2.028	0.146	0.026	7.2	1.28	0.288
P2B04	2020.1-3	5	2	2	0	1.1985	1.406	0.084	0.184	5.97	13.09	0.086
P2B05	2020.1-3	3	1	1	0	1.3709	2.219	0.108	0.005	4.87	0.23	0.59
P2B06	2020.1-3	3	1	1	0	1.3312	2.295	0.101	0.02	4.4	0.87	0.719
P2C01	2020.1-3	1	0	0	0	1.2908	1.863	0.061	0.011	3.27	0.59	1.317
P2C02	2020.1-3	1	0	0	0	1.2358	0.783	0.033	0.006	4.21	0.77	0.189
P2C03	2020.1-3	3	1	1	0	1.1024	1.056	0.051	0.077	4.83	7.29	0.029
P2C04	2020.1-3	1	0	0	0	1.1235	0.771	0.059	0.003	7.65	0.39	0.062
P2C06	2020.1-3	1	0	0	0	1.1391	0.434	0.017	0.022	3.92	5.07	0.319
P3A01	2020.1-3	9	4	4	0	1.4482	8.807	0.324	0.407	3.68	4.62	0.941
P3A03	2020.1-3	9	4	4	0	1.2184	2.969	0.168	0.184	5.66	6.2	0.134
P3A06	2020.5-6	7	3	3	0	1.2606	5.067	0.258	0.497	5.09	9.81	0.343
P3B01	2020.5-6	13	6	6	0	1.425	15.249	0.491	0.443	3.22	2.91	0.722
P3B02	2020.5-6	26	14	13	1	1.3941	14.301	0.596	0.621	4.17	4.34	0.648
P3B03	2020.5-6	1	0	0	0	1.1476	5.138	0.113	0.144	2.2	2.8	1.262
P3B04	2020.5-6	5	2	2	0	1.2853	7.5	0.285	0.326	3.8	4.35	0.751
P3B05	2020.5-6	11	5	5	0	1.2433	10.641	0.357	0.339	3.35	3.19	0.897
P3B06	2020.5-6	1	0	0	0	1.3334	9.689	0.194	0.292	2	3.01	1.509
P3C01	2020.5-6	7	3	3	0	1.3158	4.931	0.196	0.198	3.97	4.02	0.456
P3C02	2020.5-6	7	3	3	0	1.327	2.232	0.172	0.218	7.71	9.77	0.019
P3C03	2020.5-6	5	2	2	0	1.311	3.177	0.204	0.56	6.42	17.63	0.03
P3C04	2020.5-6	1	0	0	0	1.2412	1.512	0.067	0.13	4.43	8.6	0.022
P3C05	2020.5-6	5	2	2	0	1.4623	3.064	0.117	0.267	3.82	8.71	0.047
P3C06	2020.5-6	14	6	4	2	1.3026	7.456	0.254	0.273	3.41	3.66	0.739
P3D01	2020.5-6	3	1	0	1	1.1837	3.164	0.141	0.169	4.46	5.34	0.905
P3D02	2020.5-6	12	6	6	0	1.4461	6.055	0.211	0.18	3.48	2.97	0.513
P3D03	2020.5-6	6	2	1	1	1.3694	2.285	0.12	0.221	5.25	9.67	0.406
P3D04	2020.5-6	3	1	1	0	1.2369	2.593	0.098	0.287	3.78	11.07	0.023
P3D05	2020.5-6	7	3	3	0	1.4321	3.013	0.128	0.236	4.25	7.83	0.409
P3D06	2020.5-6	11	5	4	1	1.4048	7.358	0.178	0.165	2.42	2.24	0.997
P4A01	2020.5-6	7	4	4	0	1.5722	7.253	0.315	0.314	4.34	4.33	0.991
P4A02	2020.5-6	1	0	0	0	1.0122	8.243	0.097	0.136	1.18	1.65	0.003
P4B01	2020.5-6	6	3	3	0	1.4406	3.205	0.134	0.254	4.18	7.93	1.073
P4B02	2020.5-6	7	3	3	0	1.3531	6.079	0.151	0.147	2.48	2.42	0.745
P4B03	2020.5-6	3	1	1	0	1.2551	1.255	0.077	0.143	6.14	11.39	0.431
P4B04	2020.5-6	1	0	0	0	1.2092	1.296	0.088	0.134	6.79	10.34	0.344
P4B05	2020.5-6	1	0	0	0	1.3661	6.696	0.18	0.146	2.69	2.18	1.108
P4B06	2020.5-6	8	3	1	1	1.2011	5.88	0.201	0.2	3.42	3.4	1.747
P4C01	2020.5-6	16	8	7	1	1.4129	11.089	0.277	0.47	2.5	4.24	2.173
P4C02	2020.5-6	1	0	0	0	1.0649	1.136	0.084	0.122	7.39	10.74	0.385
P4C04	2020.5-6	5	2	2	0	1.3569	2.918	0.108	0.157	3.7	5.38	0.144
P4C05	2020.5-6	9	4	4	0	1.2971	3.595	0.117	0.167	3.25	4.65	0.687

Cell viability and mortality was evaluated using the WST-8 assay kit and the LDH assay kit, respectively.

ND: not determined

Supplementary Table 7. Spearman's rank correlation coefficient comparing RNAseq samples by gene expression values.

	M_0	M_1	M_2	M_3	W_4	W_0	W_1	W_2
M_0								
M_1	0.9435							
M_2	0.9452	0.9493						
M_3	0.9466	0.9417	0.9451					
M_4	0.9475	0.947	0.9503	0.9529				
W_0	0.9334	0.9254	0.9308	0.9328	0.932			
W_1	0.9311	0.9248	0.9309	0.9327	0.9326	0.9398		
W_2	0.9331	0.927	0.9352	0.9381	0.9375	0.9403	0.9451	
W_3	0.9363	0.9319	0.9346	0.9379	0.9364	0.9409	0.9427	0.9465

Supplementary Table 8. Enriched Gene Ontology terms in the differentially expressed genes in *S. proliferum* Medusa worms.

GO ID	over represented p-value	over represented adjusted p-value (FDR)	number of differentially expressed genes in this category	number of genes in this category	GO Term	genes
Unregulated genes						
GO:0004222	3.68E-21	9.53E-18	18	40	MF metalloendopeptidase activity	SprJv2_0661300.1, SprJv2_0684600.1, SprJv2_0684700.1, SprJv2_0740100.1, SprJv2_0740400.1, SprJv2_1053400.1, SprJv2_1267500.1, SprJv2_1267700.1, SprJv2_1267800.1, SprJv2_1515500.1, SprJv2_1515700.1, SprJv2_1546200.1, SprJv2_1742100.1, SprJv2_1742200.1, SprJv2_1893700.1, SprJv2_1912600.1, SprJv2_2042200.1, SprJv2_2062100.1
GO:0006508	3.31E-11	4.29E-08	26	265	BP proteolysis	SprJv2_0657500.1, SprJv2_0661300.1, SprJv2_0684600.1, SprJv2_0684700.1, SprJv2_0740100.1, SprJv2_0740400.1, SprJv2_0961100.1, SprJv2_1053400.1, SprJv2_1267500.1, SprJv2_1267700.1, SprJv2_1267800.1, SprJv2_1332400.1, SprJv2_1344500.1, SprJv2_1515500.1, SprJv2_1515700.1, SprJv2_1536900.1, SprJv2_1538300.1, SprJv2_1546200.1, SprJv2_1742100.1, SprJv2_1742200.1, SprJv2_1893700.1, SprJv2_1912600.1, SprJv2_1928100.1, SprJv2_1986000.1, SprJv2_2042200.1, SprJv2_2062100.1
Downregulated genes						
GO:0008235	1.12E-07	0.000289581	5	14	MF metalloexopeptidase activity	SprJv2_1365900.1, SprJv2_1366000.1, SprJv2_1366100.1, SprJv2_1366200.1, SprJv2_2106900.1
GO:0004177	6.96E-07	0.000821212	5	19	MF aminopeptidase activity	SprJv2_1365900.1, SprJv2_1366000.1, SprJv2_1366100.1, SprJv2_1366200.1, SprJv2_2106900.1
GO:0030145	9.52E-07	0.000821212	5	21	MF manganese ion binding	SprJv2_1365900.1, SprJv2_1366000.1, SprJv2_1366100.1, SprJv2_1366200.1, SprJv2_2106900.1
GO:0006508	4.67E-06	0.003019298	12	265	BP proteolysis	SprJv2_0400500.1, SprJv2_1365900.1, SprJv2_1366000.1, SprJv2_1366100.1, SprJv2_1366200.1, SprJv2_1536700.1, SprJv2_1738200.1, SprJv2_1860200.1, SprJv2_1900500.1, SprJv2_1927000.1, SprJv2_2048200.1, SprJv2_2106900.1
