# Supplementary material for: Genome of the fatal tapeworm Sparganum proliferum uncovers mechanisms for cryptic life cycle and aberrant larval proliferation
Source: Commun Biol. 2021 May 31;4:649. doi: 10.1038/s42003-021-02160-8 (PMC8166898; doi:10.1038/s42003-021-02160-8)
Supplement: Supplementary file 2 — Description of Additional Supplementary Files [file 42003_2021_2160_MOESM2_ESM.pdf]

Description of Additional Supplementary Files

File name: Supplementary Data

Description:

Supplementary Data 1: Eukaryotic core genes (CEGs) not detected in the tapeworm species by CEGMA analyses. "1" and "0" indicates presence and absence of the CEG, respectively.

Supplementary Data 2: Numbers of Pfam domains observed in the S. proliferum and S. erinaceieuropaei

genomes.

Supplementary Data 3: Significantly Expanded Gene Families in S. proliferum lineage.

Supplementary Data 4: Significantly Contracted Gene Families in S. proliferum lineage.

Supplementary Data 5: Significantly Expanded Gene Families in Spirometra erinaceieuropaei lineage.

Supplementary Data 6: Significantly Contracted Gene Families in Spirometra erinaceieuropaei lineage.

Supplementary Data 7: Orthologue inferences in S. proliferum and S. erinaceieuropaei for main genes that regulate development in animals.

Supplementary Data 8: Putatively horizontally transferred genes in S. proliferum, supported by lineage probability index (LPI).

Supplementary Data 9: Putatively horizontally transferred genes in S. erinaceieuropaei, supported by

lineage probability index (LPI).

Supplementary Data 10: Single copy gene set used for Positive/Relax Selection Scans (dN/dS)

Supplementary Data 11: Differentially expressed genes in highly branching worms (Medusa form) relative to static worms (Wasabi form) in S. proliferum. Transposon proteins were shown in gray.
